# Role of ABCB1 and ABCB4 in renal and biliary excretion of perfluorooctanoic acid in mice

**DOI:** 10.1265/ehpm.23-00284

**Published:** 2024-03-22

**Authors:** Kazuyoshi Furukawa, Kahori Okamoto-Matsuda, Kouji H. Harada, Mutsuko Minata, Toshiaki Hitomi, Hatasu Kobayashi, Akio Koizumi

**Affiliations:** 1 Department of Health and Environmental Sciences, Kyoto University Graduate School of Medicine, Yoshida Konoe, Sakyo, Kyoto 606-8501, Japan; 2 Department of Preventive Medicine, St. Marianna University School of Medicine, Kawasaki 216-8511, Japan; 3 Department of Environmental and Molecular Medicine, Mie University Graduate School of Medicine, Mie 514-8507, Japan; 4 Public Health and Welfare Institute, Public Interest Incorporated Association Kyoto Hokenkai, Kyoto 616-8141, Japan

**Keywords:** Perfluorooctanoic acid, ABCB1, ABCB4, Renal clearance, Biliary excretion

## Abstract

**Background:**

Perfluorooctanoic acid (PFOA) is one of the major per- and polyfluoroalkyl substances. The role of ATP-binding cassette (ABC) transporters in PFOA toxicokinetics is unknown.

**Methods:**

In this study, two ABC transporters, ABCB1 and ABCB4, were examined in mice with single intravenous PFOA administration (3.13 µmol/kg). To identify candidate renal PFOA transporters, we used a microarray approach to evaluate changes in gene expression of various kidney transporters in *Abcb4* null mice.

**Results:**

Biliary PFOA concentrations were lower in *Abcb4* null mice (mean ± standard deviation: 0.25 ± 0.12 µg/mL) than in wild-type mice (0.87 ± 0.02 µg/mL). Immunohistochemically, ABCB4 expression was confirmed at the apical region of hepatocytes. However, renal clearance of PFOA was higher in *Abcb4* null mice than in wild-type mice. Among 642 solute carrier and ABC transporters, 5 transporters showed significant differences in expression between wild-type and *Abcb4* null mice. These candidates included two major xenobiotic transporters, multidrug resistance 1 (*Abcb1*) and organic anion transporter 3 (*Slc22a8*). *Abcb1* mRNA levels were higher in *Abcb4* null mice than in wild-type mice in kidney. In *Abcb4* null mice, *Abcb1b* expression was enhanced in proximal tubules immunohistochemically, while that of *Slc22a8* was not. Finally, in *Abcb1a/b* null mice, there was a significant decrease in the renal clearance of PFOA (0.69 ± 0.21 vs 1.1 mL ± 0.37/72 h in wild-type mice). A homology search of ABCB1 showed that several amino acids are mutated in humans compared with those in rodents and monkeys.

**Conclusions:**

These findings suggest that, in the mouse, *Abcb4* and *Abcb1* are excretory transporters of PFOA into bile and urine, respectively.

**Supplementary information:**

The online version contains supplementary material available at https://doi.org/10.1265/ehpm.23-00284.

## Graphical Abstract

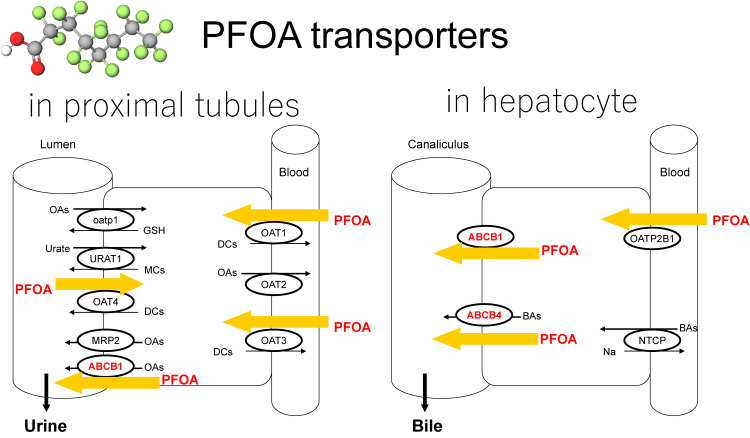

## Introduction

Environmental pollutions due to industrial and economic activities have posed various health risks [1–3]. Per- and polyfluoroalkyl substances (PFASs) have been used since approximately the 1940s as water and oil repellents, aqueous film-forming foam as a fire extinguisher, and surfactants [4]. PFASs are man-made chemicals with a structure in which the hydrogen on the carbon chain is replaced by fluorine atoms. Therefore, many PFASs are chemically stable substances, and even if they are partially metabolized or decomposed, they become stable end products and are believed to remain in the environment for a long period. The Organization for Economic Cooperation and Development estimated that there are more than 4,730 chemicals that are in the PFAS category [5].

Among the PFASs, perfluorooctanoic acid (PFOA) is mainly used as a processing aid for fluoropolymer and surfactant production [6]. Contamination of PFOA around fluoropolymer manufacturing plants has been reported [7–9]. PFOA poses particular public health concerns because of its long-term persistence and bioaccumulation in the environment and in humans [10–13]. The production of PFOA is internationally regulated under the Stockholm Convention [14].

The effects of PFOA on health have been reported. The International Agency for Research on Cancer currently lists PFOA as Group 1 (carcinogenic to humans) [15]. Tumors of the liver, mammary gland, testis, and pancreas have been observed in rodents, and epidemiological studies have suggested an association between PFOA exposure and kidney and testicular cancers [16]. The C8 Science Panel, which is an independent panel of scientists, conducted a survey of 69,000 residents as a result of a settlement between DuPont and residents in Ohio and West Virginia, USA. The panel ultimately concluded that there are “probable links” between PFOA exposure and high cholesterol concentrations, kidney cancer, testicular cancer, thyroid disease, ulcerative colitis, and gestational hypertension [16]. The association between PFOA exposure and kidney cancer was confirmed independently in a nested case–control study from the Prostate, Liver, Colorectal, Ovarian (PLCO) Trial [17].

The cause of PFOA’s extremely long half-life in humans is not well understood. An epidemiological study of retired workers from a PFOA production plant operated by 3M showed that the plasma elimination half-life was 3.8 years [18]. In contrast, the plasma elimination half-life of PFOA is much shorter in mice (15–20 days) [19], rats (<1–15 days) [20], and cynomolgus monkeys (20–35 days) than in humans [21]. The reason for the long half-life of PFOA in humans is because of the virtual absence of renal excretion [22], while biliary excretion of PFOA is high compared with renal excretion [23, 24]. Several organic anion transporters (solute carrier family 22, member 6, [*SLC22A6*] and *SLC22A8*) transport PFOA in rats and humans, but cannot explain the low excretion rate in the kidney because there is no significant difference in their activities [25]. *SLC22A11* encoding human OAT4 also transports PFOA *in vitro* [26], which may confer the placental barrier. Human intestinal Caco-2 cells show uptake of different PFASs, including PFOA, suggesting involvement of organic anion transporting polypeptides such as OATP2B1 [27, 28]. In addition, Na^+^/taurocholate cotransporting polypeptide (SLC10A1) and apical sodium-dependent bile acid transporter (SLC10A2) mediate uptake of PFASs into hepatocytes [29]. In our previous study [30], biliary excretion of PFOA was associated with the expression of ATP-binding cassette (ABC) sub-family B, member 4 (*Abcb4*: *Mdr2*) in the liver of mice. However, there have been few investigations on active transporters of PFASs *in vivo*. In this study, we aimed to examine two ABC transporters, ABCB1 and ABCB4, for biliary and renal excretion of PFOA in knockout mice.

## Material and methods

### Animals

Animal studies, including animal care and all experimental procedures, were in accordance with the Animal Welfare Guidelines of Kyoto University. All experimental procedures were reviewed and approved by the Kyoto University Animal Research Committee (approval number: MedKyo11069). Wild-type mice (FVB) and *Abcb4* null mice (FVB.129P2-*Abcb4^tm1Bor^*/J, FVB *Abcb4^(−/−)^*) were purchased from The Jackson Laboratory (Bar Harbor, ME, USA). *Abcb1a/1b* null mice (FVB.129P2-*Abcb1a^tm1Bor^ Abcb1b^tm1Bor^*/N12, FVB *Mdr1a/1b^(−/−)^*) were purchased from Taconic Farms (Germantown, NY, USA). The mice were housed in the Kyoto University Institute of Laboratory Animals. A standard commercial lab chow diet (F-2, 3.73 kcal/g; Funahashi Farm Corp., Chiba, Japan) was used. All mice were maintained at an ambient temperature of 24 °C ± 2 °C and 50% ± 10% humidity with a 12 h dark-light cycle (lights on at 7:00 a.m.).

### Experimental design

All experiments were performed with male mice aged 8–10 weeks (25–30 g). The number of mice in each group ranged from 3 (*Abcb4* KO/WT groups) to 5 (*Abcb1a/b* KO/WT groups). Mice were intravenously administered PFOA as a single dose (dose: 3.13 µmol/kg, injection volume: 10 mL/kg).

We conducted two kinetic studies. The first study was conducted to observe the time course of plasma PFOA concentrations. In this experiment, blood was collected at 0, 2, 24, and 72 h after PFOA administration. Mice were killed by cervical dislocation under sevoflurane anesthesia after observation for 72 h. At sacrifice, tissue samples and blood were collected. Blood samples were centrifuged to separate plasma at 800 × *g*. The remaining carcass was flash-frozen in liquid nitrogen and stored at −80 °C.

In the second experiment, we determined renal clearance. Urine was collected at 0, 24, 48, and 72 h after PFOA administration, and blood and bile were collected at the sacrifice. To calculate renal clearance of PFOA (CL_r_), we used the following equation:

$$\begin{array}{rl} & {\text{CL}}_{r}\text{ of PFOA (mL/observed duration)}=\\ & \;\frac{\text{cumulative amount of PFOA in urine (}\mu \text{g/observed duration)}}{\text{PFOA concentration in plasma at 72}\;\text{h (}\mu \text{g/mL)}} \end{array}$$

### Determination of PFOA concentrations in biological samples

In a polypropylene tube, diluted plasma, urine, or homogenized kidney was combined with 10 µL of a 1 µg/mL solution of ^13^C_2_-PFOA as an internal standard (donated by the Environmental Protection Agency of the USA, originally synthesized by Perkin Elmer, Boston, MA, USA). A volume of 1 mL of tetrabutylammonium hydrogen sulfate was added to 2 mL of 0.5 M sodium carbonate buffer solution (pH adjusted to 10) and vortexed, and then 2 mL of methyl *tert*-butyl ether was added and vortexed [13]. The tube was centrifuged to separate the aqueous and organic phases, and the methyl *tert*-butyl ether layer was transferred to a glass tube and evaporated to dryness under a gentle stream of dry nitrogen. The residue was then re-dissolved in 100 µL of 100 mM benzyl bromide acetone and was heated for 1 hour at 60 °C in an autosampler vial. Derivatized extracts were analyzed using gas chromatography-mass spectrometry (Agilent 6890GC/5973MSD; Agilent Technologies Japan, Tokyo, Japan) in the electron impact ionization mode [31]. Benzylated PFOA was separated on a DB-5MS column (30 m length, 0.25 mm inner diameter, 1 µm film thickness) with helium carrier gas. Splitless injections (2 µL) were performed with the injector set at 220 °C, and the split was opened after 1.5 min. The initial oven temperature was 70 °C for 1.5 min, increased by 15 °C min^−1^ to 100 °C, and then increased by 40 °C min^−1^ to 240 °C. The ion fragment (m/z 504, [M]^+^) was monitored and used as quantification ions. The detection limit was 2 ng/g of sample. The mean recovery rate of ^13^C_2_ PFOA from samples was 93.1% ± 8.3%.

### Microarray analysis of gene expression

Kidneys were collected from *Abcb4* null mice and wild-type mice (3 mice aged 8–10 weeks in each group). Approximately 30 mg of kidney was diced on ice, and then total RNA was extracted using an RNeasy Lipid Tissue Mini Kit (Qiagen, Tokyo, Japan). The concentration and purity of total RNA were measured using a NanoDrop ND-1000 spectrophotometer (NanoDrop Technologies, Wilmington, DE, USA) and an Agilent 2100 Bioanalyzer (Agilent Technologies Japan), respectively [32]. Extracted RNA was stored at −80 °C until analysis. Cyanine-labeled cDNA was synthesized from the total RNA samples and hybridized to oligo DNA microarray slides (Whole Mouse Genome 4x44k; Agilent Technologies Japan). The slides were scanned with an Agilent GeneArray Scanner (Agilent Technologies Japan). Information from probe features was obtained using Feature Extraction software version 9.5 (Agilent Technologies Japan). Raw and processed data and the experimental design have been submitted to the NCBI Gene Expression Omnibus according to the MIAME code, and are available at http://www.ncbi.nlm.nih.gov/geo/ (GEO accession number: GSE30084).

### Sequence homology analysis

The following protein sequences were analyzed: human ABCB1 (NCBI: NP_000918.2), rhesus monkey ABCB1 (AAN07779.1), rat Abcb1b (NP_036755.3), and mouse Abcb1b (NP_035205.1). Identities of sequences were evaluated using BLAST (ver. 2.14.0) (https://blast.ncbi.nlm.nih.gov/). Sequence alignment was performed using ClustalW (ver. 2.1) [33] and Jalview (ver. 2.11.2.0) [34].

### Statistical analysis

Three to five animals were used in each group. All results are expressed as the mean ± standard deviation. Comparisons between two groups were performed using the unpaired Student *t*-test. A probability of <0.05 was considered statistically significant. The analyses were performed using JMP Pro Statistical Software, version 16 (SAS institute, Cary, NC, USA).

## Results and discussion

### Kinetics of PFOA uptake and excretion in wild-type and *Abcb4* null mice

**Fig. 1:**
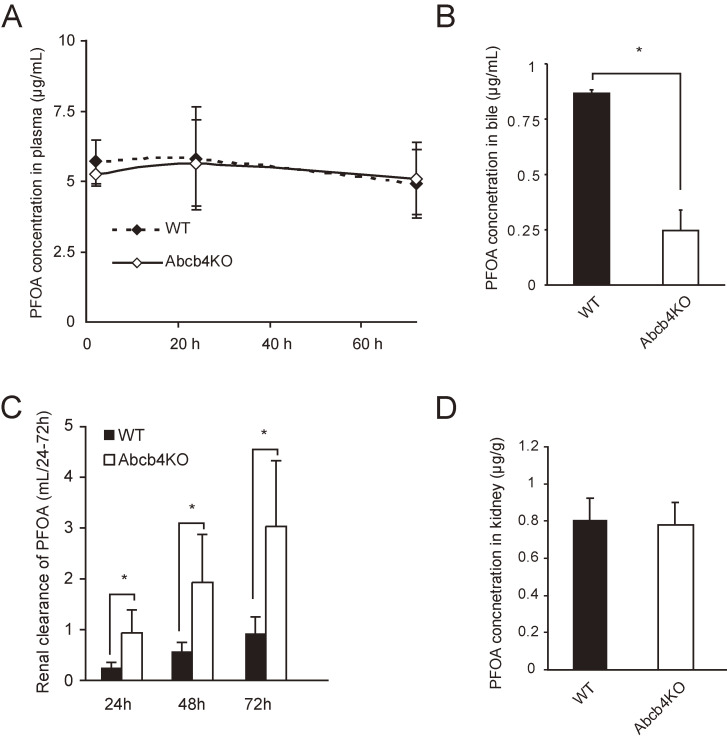
Excretion and distribution of perfluorooctanoic acid (PFOA) in wild-type and *Abcb4* null mice. (A) Plasma concentrations of PFOA in mice at 2, 24, and 72 h after intravenous administration. (B) PFOA concentrations in bile at 72 h. (C) Cumulative CL_r_ of PFOA (mL/24–72 h) at 24, 48, and 72 h after intravenous administration. (D) PFOA concentrations in the kidney at 72 h. Filled bars and triangles indicate wild-type (WT) mice and open ones *Abcb4* null (Abcb4KO) mice. Results are expressed as the mean ± standard deviation. Differences in mean values between the two groups were examined using Student’s *t*-test (* indicates *p* < 0.05).

There was no significant difference in plasma PFOA concentrations between wild-type and *Abcb4* null mice during 72 h after PFOA administration (Fig. 1A). PFOA concentrations in plasma did not significantly change from 2 to 72 h (Fig. 1A). Therefore, for subsequent analyses, we used plasma PFOA concentrations at 72 h.

Biliary PFOA concentrations after single intravenous administration (3.13 µmol/kg) were lower in *Abcb4* null mice (mean: 0.25 ± 0.12 µg/mL) than in wild-type mice (0.87 ± 0.02 µg/mL) (Fig. 1B, p < 0.05 by Student’s *t*-test). We previously observed that an absence of *Abcb4* induction in PPARα null mice significantly decreased biliary PFOA excretion [30]. Immunohistochemistry also showed ABCB4 expression in the liver in wild-type mice (Fig. S1). Taken together, these results suggest that *Abcb4* is a transporter of PFOA from hepatocytes into bile.

The mean CL_r_ of PFOA in wild-type mice was 0.93 ± 0.32 mL/72 h (Fig. 1C), while that in *Abcb4* null mice was significantly higher (3.03 ± 1.3 mL/72 h, p < 0.05 by Student’s *t*-test). Higher renal excretion rates were consistently observed 24 and 48 h after PFOA administration. PFOA content in the kidney of wild-type mice was similar to that in *Abcb4* null mice (Fig. 1D). Immunohistochemistry showed a low level of ABCB4 protein in the wild-type kidney (Fig. S1), suggesting that *Abcb4* is not directly involved in an increased CL_r_ after its gene ablation.

### mRNA profiles in the kidney

To identify a candidate PFOA transporter, we compared kidney mRNA levels by microarray analysis in wild-type and *Abcb4* null mice (Table S1). The candidate transporter may function in excretion or absorption processes. Therefore, we introduced the criterion that a gene showing upregulation or downregulation by a significant (p < 0.05) change of at least 1.5-fold could be considered as a candidate.

**Fig. 2:**
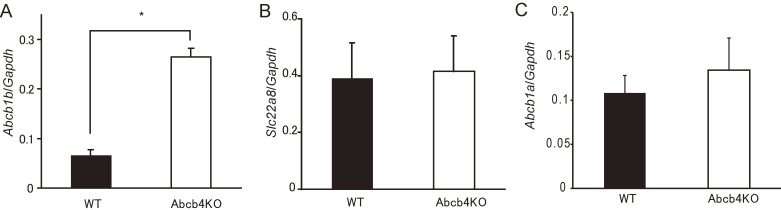
mRNA expression of transporters in kidney tissue of wild-type and *Abcb4* null mice. Results of quantitative PCR for *Abcb1b* (A), *Slc22a8* (B), and *Abcb1a* (C) are presented as the means ± standard deviation (n = 5). Filled bars indicate wild-type (WT) mice and open ones *Abcb4* null (Abcb4KO) mice. Expression of transporters was standardized to that of *Gapdh*. * indicates *p* < 0.001 using Student’s *t*-test.

Screening of 94 ABC transporters and 548 solute carrier family transporters showed several candidate genes (Table S2). The upregulated genes were *Abcb1b*, *Slc6a15*, and *Slc22a7* (all p < 0.05 by Student’s *t*-test), while *Slc7a12* and *Slc22a8* were downregulated (both p < 0.05 by Student’s *t*-test). Of these candidate genes, *Slc22a7*, *Slc22a8*, and *Abcb1b* are involved in xenobiotic transport [35], although *Slc22a7* does not have transport activity for PFOA [25, 36]. Therefore, we chose two genes for further study, namely *Slc22a8* and *Abcb1b*. Quantitative real-time polymerase chain reaction (PCR) in *Abcb4* null mice showed upregulation of *Abcb1b* mRNA, but failed to show downregulation of *Slc22a8* mRNA (Fig. 2A, B), which is consistent with results from a previous report [37]. *Abcb1a* is a paralog of *Abcb1b*. Therefore, we investigated possible induction of *Abcb1a* in *Abcb4* null mice by real-time PCR, but failed to detect any such induction (Fig. 2C).

### Immunohistochemistry of ABCB1b and SLC22A8 in the kidney

**Fig. 3:**
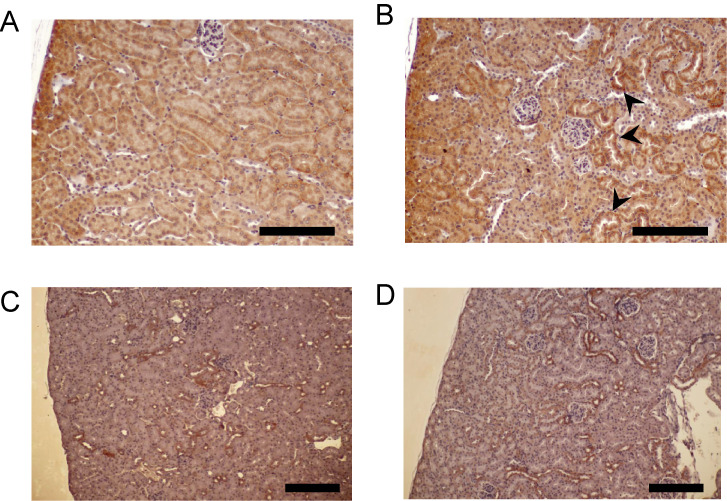
Immunohistochemical detection of ABCB1b (A, B) and SLC22A8 (C, D) in mouse kidney. Sections of kidney were analyzed by DAB-staining (red color) followed by nuclear counterstaining with hematoxylin. (A, C) Cortex in wild-type mice, (B, D) cortex in *Abcb4*-null mice. Arrows indicate ABCB1 positive tubules. Scale bars, 500 µm.

ABCB1b was localized at the brush border of proximal tubules in wild-type mice (Fig. 3A). In *Abcb4* null mice, ABCB1b was overexpressed in the proximal tubules (Fig. 3B). SLC22A8 was expressed mainly in the basolateral membrane of the proximal tubules in wild-type and *Abcb4* null mice, but its expression level was not different between wild-type and *Abcb4* null mice (Fig. 3C, D). These data support the hypothesis that ABCB1b transports PFOA.

### Pharmacokinetics of PFOA in *Abcb1a/1b* null mice

**Fig. 4:**
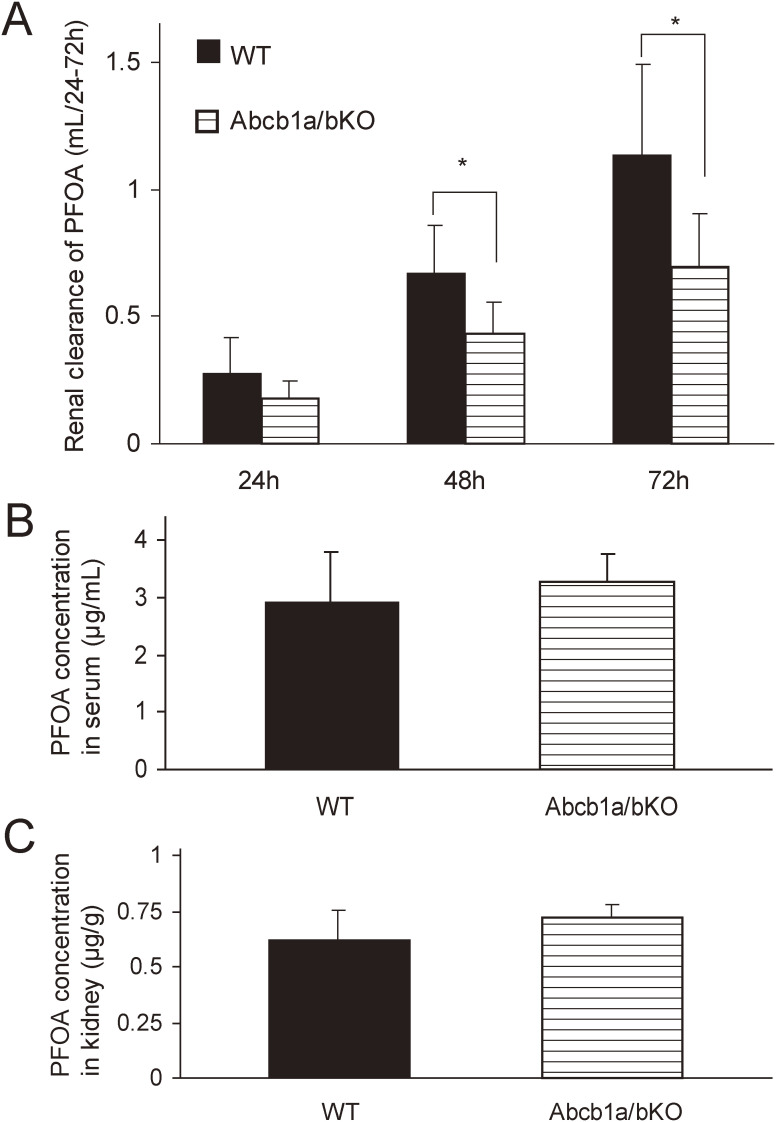
Excretion and distribution of perfluorooctanoic acid (PFOA) in wild-type and *Abcb1a/1b* null mice. (A) Cumulative CL_r_ of PFOA (mL/24–72 h) at 24, 48, and 72 h after intravenous administration. (B) Plasma concentrations of PFOA (µg/mL) at 72 h after intravenous administration. (C) PFOA concentrations in the kidney (µg/g) at 72 h. Filled bars indicate wild-type (WT) mice and dashed ones *Abcb1a/b* null (Abcb1a/bKO) mice. Results are expressed as the mean ± standard deviation. Differences in mean values between the two groups were examined using Student’s *t*-test (* indicates *p* < 0.05).

We next conducted a kinetic study using *Abcb1a/1b* null mice to test the hypothesis of whether ABCB1b transports PFOA in the kidney. *Abcb1a/1b* null mice showed a significantly lower CL_r_ of PFOA than wild-type mice (0.432 ± 0.127 vs 0.675 ± 0.181 mL/48 h and 0.694 ± 0.209 vs 1.138 ± 0.354 mL/72 h, respectively; both p < 0.05 by Student’s *t*-test, Fig. 4A). Plasma and kidney tissue levels of PFOA did not change in *Abcb1a/b* null mice compared with those in wild-type mice (Fig. 4B, C), indicating that *Abcb1a/1b* ablation disrupted the secretion of PFOA from the kidney.

### Sequence conservation of ABCB1 between species

Renal excretion of PFOA in humans is markedly lower than that in other species, such as mouse, rat, and monkey [22]. This finding suggests that species differences in the *ABCB1* sequence could be associated with substrate affinity. Therefore, we evaluated the sequence conservation of ABCB1 between these species. Mice and rats have two *Abcb1* paralogs *Abcb1a* and *Abcb1b*, while monkeys and humans do not have two paralog genes. In this study, the sequence of mouse/rat *Abcb1b* and human/monkey *ABCB1* were analyzed because we speculated that *Abcb1b* contributes to renal secretion of PFOA in mice.

**Fig. 5:**
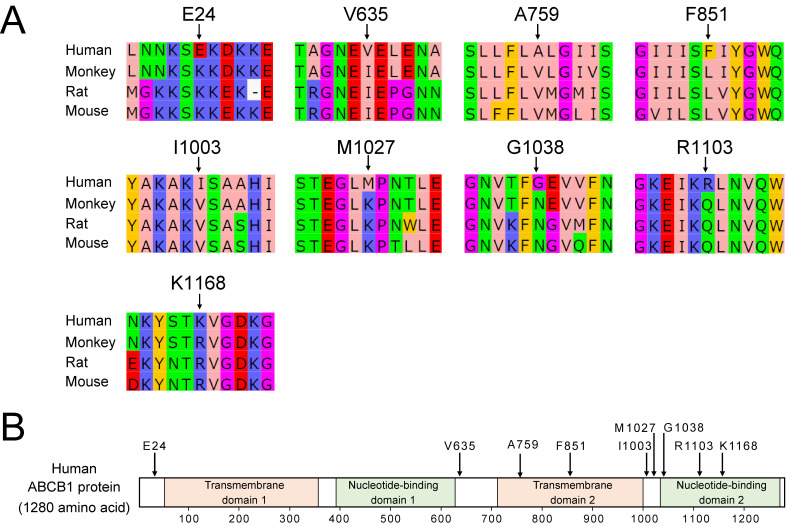
Non-conserved amino acids in human ABCB1 compared with monkey ABCB1 and rat/mouse Abcb1b. (A) Sequence alignment among four species (human, monkey, rat and mouse). Sequence alignment was performed using ClustalW and Jalview. The amino acids are colored according to “Zaapo”, which is the color scheme of Jalview. (B) Positions of non-conserved amino acids and functional domains of human ABCB1.

Protein sequence identities of monkey ABCB1, rat ABCB1b, and mouse ABCB1b were 96%, 81% and 81%, respectively, compared with human ABCB1 (Table S3). We searched amino acids that are conserved between the monkey, rat, and mouse, but not in humans, by sequence homology analysis. Nine amino acids (E24, V635, A759, F851, I1003, M1027, G1038, R1103, and K1168) were identified in human ABCB1 (Table S4 and Fig. 5A). As a result, among the nine non-conserved amino acids, A759 and F851 are present in the transmembrane domain, and G1038, R1103, and K1168 are present in the ATP-binding domain (Fig. 5B). The lack of conservation of these amino acids may affect human ABCB1 function. ABCB1 has a wide range recognition of substrates and it differs among species [38]. These non-conserved amino acids might reduce the PFOA-secreting function of human ABCB1 and *in vitro* site-directed mutagenesis experiments will elucidate the mechanisms in substrate recognition.

### Potential transporters of PFOA

In this study, we found that *Abcb4* was a transporter of PFOA from blood into bile as suggested by our previous study [30]. Intriguingly, we found that *Abcb1b* transported PFOA into urine in mice. The contribution of *Abcb1b* to the renal excretion of PFOA under physiological conditions was estimated to be approximately 30%. ABCB1b is localized at the apical membrane in renal tubules where it transports various drugs to the lumen [39]. To the best of our knowledge, *Abcb1b* is the first transporter of PFOA that has been located to the apical membrane.

The biological half-life of PFOA in humans (3.8 years) [18] can be explained by a low excretion rate in the kidney [22] and enterohepatic circulation of PFOA [23, 24]. The low urinary excretion rate may be explained by the lower substrate specificity of human ABCB1 to PFOA than that in other species. In contrast, the excretion rate of PFOA into bile is 30- to 60-fold larger than the urinary excretion rate [23, 24]. Therefore, enhancing fecal elimination by decreasing the reabsorption rate in the enterohepatic circulation of PFOA may shorten the biological half-life in humans. Several drugs have been developed to enhance the elimination of bile into feces [40, 41], providing a pharmacological approach to enhance the elimination of PFOA and other PFASs from the body. In fact, cholestyramine, bile acid sequestrant resin, is known to enhance fecal excretion of PFAS through increase of bile acid in feces [42].

### Limitations in this study

Renal clearance of PFOA in *Abcb1a/b*-null mice was decreased compared with WT mice. Ablations of *Abcb1a/b* genes may cause off-target effects in gene expression in kidney as observed in *Abcb4* ablation while there have been no reports on significant compensation effects of *Abcb1a/b* deletion. If the potentially induced off-target gene may affect the renal excretion change more than 30%, contribution of ABCB1 may decline. Confirmation of gene expression in the tissue in mice and *in vitro* experiments of transporting activity of ABCB1 for PFOA will support this study results.

As we investigated before, solute carrier transporters are involved in PFOA kinetics [25]. Further, reported biological half-lives of PFOA significantly differ from humans [11]. Protein bindings of PFOA to serum proteins may further modify the kinetics. The relative contribution to ABC transporters should be evaluated in experimental animals and humans in future.

## Conclusion

This study shows that ABCB4 is one of the major transporters of PFOA in the bile; and ABCB1 is one of the major transporters of PFOA in kidney in the mouse. The human ABCB1 system may not have substrate affinity to PFOA as Abcb1a/1b does in the rodent, which could explain the much lower renal clearance of PFOA in humans than in rodents. In contrast, the human *ABCB4* system, the synteny of mouse *Abcb4* in rodents, is involved in excretion into bile in humans and rodents [24]. Further studies on transporters are required to establish not only rational risk assessment of PFAS, but also to develop pharmacological interventions to enhance the elimination of PFASs from the body.

## Abbreviations

ABC: ATP-binding cassette
PCR: polymerase chain reaction
PFOA: Perfluorooctanoic acid
PFASs: Per- and polyfluoroalkyl substances
PLCO trial: Prostate, Liver, Colorectal, Ovarian (PLCO) trial

## Supplementary information

**Additional file 1: Supplemental description of methods.** **Table S1.** Results of microarray analysis for 642 potential transporter genes. **Table S2.** Microarray analysis for expression of transporter in mouse kidney. **Table S3.** Sequence identity between human, monkey, rat and mouse ABCB1. **Table S4.** Non-conserved amino acids in human ABCB1. **Figure S1.** Immunohistochemical analysis of ABCB4 expression in wild-type mouse kidney and liver.

## Declarations

### Animal welfare

Animal studies, including animal care and all experimental procedures, were in accordance with the Animal Welfare Guidelines of Kyoto University. All experimental procedures were reviewed and approved by the Kyoto University Animal Research Committee (approval number: MedKyo11069).

### Data availability statement

The data that support the findings of this study are available from the corresponding author upon reasonable request. Raw and processed data and the experimental design of microarray analysis are available at http://www.ncbi.nlm.nih.gov/geo/ (GEO accession number: GSE30084).
