# Supplementary material for: Role of ABCB1 and ABCB4 in renal and biliary excretion of perfluorooctanoic acid in mice
Source: Environ Health Prev Med. 2024 Mar 22;29:21. doi: 10.1265/ehpm.23-00284 (PMC10965415; doi:10.1265/ehpm.23-00284)
Supplement: Supplementary file 1 — Additional file 1: Supplemental description of methods. Table S1. Results of microarray analysis for 642 potential transporter genes. Table S2. Microarray analysis for expression of transporter in mouse kidney. Table S3. Sequence identity between human, monkey, rat and mouse ABCB1. Table S4. Non-conserved amino acids in human ABCB1. Figure S1. Immunohistochemical analysis of ABCB4 expression in wild-type mouse kidney and liver. [file ehpm-29-021-s001.pdf]

Supplemental materials

Role of ABCB1 and ABCB4 in Renal and Biliary Excretion of Perfluorooctanoic Acid in Mice

Kazuyoshi Furukawa[#], Kahori Okamoto-Matsuda[#], Kouji H. Harada*, Mutsuko Minata, Toshiaki Hitomi, Hatasu Kobayashi and Akio Koizumi

Department of Health and Environmental Sciences, Kyoto University Graduate School of Medicine, Yoshida, Kyoto, Japan

# These authors contributed equally to this work

*Corresponding author

Kouji H. Harada

Department of Health and Environmental Sciences, Kyoto University Graduate School of Medicine, Yoshida Konoe, Sakyo, Kyoto 606-8501, Japan.

Tel: +81-75-753-4490; Fax: +81-75-753-4490

E-mail: kharada-hes@umin.ac.jp

Supplemental description of methods

Quantitative RT-PCR analysis of Abcb1b and Slc22a8.

Quantitative real-time PCR (RT-PCR) was used to measure *Abcb1b* and *Slc22a8* mRNA levels in the kidney. Total RNA was extracted as described above. Aliquots (10 ng) were amplified using QuantiTect® SYBR® Green RT-PCR (Qiagen). Quantification of the amplified products was performed on an ABI PRISM 7700 Sequence Detection System (Applied Biosystems Japan, Tokyo, Japan). The expression data from each sample were normalized to glyceraldehyde 3-phophate dehydrogenase (*Gapdh*) mRNA from the same individual sample, to correct for differences in efficiency of RNA extraction and RNA quality.

The following primers were used. For *Gapdh*: forward, 5'-ATGGTGAAGGTCGGTGTGAA -3', reverse, 5'-GAGTGGAGTCATACTGGAAC-3' (Hirosawa *et al.* 2008); for *Abcb1b* (corresponding to GenBank accession number NM_011075): forward, 5'-GGAACATTACTTCCCCTCTTG-3', reverse, 5'-GAAAGCATCAATACAGGGGGCAG-3'; for *Slc22a8* (corresponding to GenBank accession number NM_001164635.1): forward, 5'-CAGCACCAGAGACACCATTG-3', reverse, 5'-TCTCCAAACACAGGTCCTCC-3'; for *Abcb1a* (corresponding to GenBank accession number NM_011076.2): forward, 5'-GCTTTGCAAGTGTAGGAAACG-3', reverse, 5'-TCCTCCAGTTTGGCAAACATG-3'.

Immunohistochemistry for ABCB4, ABCB1B and SLC22A8.

For light microscopy, kidneys were processed by routine paraffin sectioning. To detect ABCB4 and ABCB1b, we used the same antibodies as described for western blot

analysis. Detection of these two proteins was accomplished using a mouse on mouse (MOM) peroxidase kit according to the manufacturer's protocol (Vector Laboratories, Burlingame, CA, USA). Primary anti-ABCB4 antibody (P3II-26) and anti-ABCB1b antibody (C219) were diluted (1:500 and 1:50, respectively), and applied at r.t. for 1 hour. Slides were then incubated for 1 hour with secondary biotinylated anti-mouse IgG. To detect ABCB4 and dipeptidyl peptidase IV (DPP IV, an apical membrane marker), fluorescent conjugated secondary antibody (Alexa Fluor 594 or Alexa Fluor 488, Molecular probes, Invitrogen) was applied for 30 minutes at r.t. To detect ABCB1b, ABC Elite reagent was applied for 30 minutes at r.t. Immunoreactivity was visualized using 3,3'-diaminobenzidine, followed by nuclear counterstaining with hematoxylin. Slides were mounted on glycerol gelatin slides and viewed on a non-inverted microscope using objectives of ×10, ×20, and ×40. Images were taken with an EOS kiss X3 camera, (Canon, Tokyo, Japan).

Table S1. Results of microarray analysis for 642 potential transporter genes

FeatureNum	GeneName	Description	gProcessedSignal FVB wild type 1	FVB wild type 2	FVB wild type 3	FVB ABCB4 null 1	FVB ABCB4 null 2	FVB ABC
60	Slc25a30	Mus musculus solute carrier family 25, member 30 (Slc25a30), mRNA [NM_026232]	243.1	265.4	233.5	374.7	333.5	
110	Slc35a1	Mus musculus solute carrier family 35 (CMP-sialic acid transporter), member 1 (Slc35a1), mRNA [NM_011895]	1446.9	1596.9	1777.8	1417.0	1948.5	
272	Slc30a2	Mus musculus 13 days embryo male testis cDNA, RIKEN full-length enriched library, clone:6030430C21 product:ZINC TRANSPORTER 2 (ZNT-2) homolog [Rattus norvegicus], full insert sequence [AK031425]	10883.5	13080.2	13312.4	10777.2	11829.8	
287	Slc39a13	Mus musculus solute carrier family 39 (metal ion transporter), member 13 (Slc39a13), mRNA [NM_026721]	767.9	798.1	1156.5	796.9	921.3	
493	Slc20a1	Mus musculus solute carrier family 20, member 1 (Slc20a1), mRNA [NM_015747]	4618.9	4308.7	5026.1	5028.1	5173.4	
495	Slc19a1	Mus musculus solute carrier family 19 (sodium/hydrogen exchanger), member 1 (Slc19a1), mRNA [NM_031196]	266.2	263.8	342.7	246.0	276.3	
525	Abca13	Mus musculus adult male bone cDNA, RIKEN full-length enriched library, clone:9830132L24 product:hypothetical protein, full insert sequence. [AK036546]	4.1	0.7	0.1	−0.5	−0.1	
617	Abca14	Mus musculus ATP-binding cassette, sub-family A (ABC1), member 14 (Abca14), mRNA [NM_026458]	8.6	7.1	5.5	3.2	7.4	
720	Abcd3	Mus musculus ATP-binding cassette, sub-family D (ALD), member 3 (Abcd3), mRNA [NM_008991]	55403.4	65490.2	63988.7	50461.5	65445.5	
728	Slc4a7	Mus musculus solute carrier family 4, sodium bicarbonate cotransporter, member 7 (Slc4a7), mRNA [NM_001033270]	443.7	479.6	615.4	503.7	618.7	
866	Slc9a9	Mus musculus solute carrier family 9 (sodium/hydrogen exchanger), isoform 9 (Slc9a9), mRNA [NM_177909]	5.5	5.4	4.9	12.3	8.3	
868	Slc7a15	Mus musculus solute carrier family 7 (cationic amino acid transporter, y+ system), member 15 (Slc7a15), transcript variant 2, mRNA [NM_177802]	3.2	−0.4	2.9	2.2	0.5	
962	Slc7a6	Mus musculus solute carrier family 7 (cationic amino acid transporter, y+ system), member 6 (Slc7a6), mRNA [NM_178798]	51.1	55.5	52.1	57.8	51.8	
983	Slc25a46	Mus musculus 10 days neonate cerebellum cDNA, RIKEN full-length enriched library, clone:6530437D17 product:similar to TB1 PROTEIN (FRAGMENT) [Homo sapiens], full insert sequence. [AK078383]	1.7	1.2	2.8	2.1	2.5	
1048	Slc26a5	Mus musculus solute carrier family 26, member 5 (Slc26a5), mRNA [NM_030727]	4.3	3.5	9.0	3.2	2.4	
1099	Slc22a17	Mus musculus solute carrier family 22 (organic cation transporter), member 17 (Slc22a17), mRNA [NM_021551]	283.9	325.7	398.8	307.8	324.5	
1114	Slc25a41	Mus musculus solute carrier family 25, member 41 (Slc25a41), mRNA [NM_175333]	9.7	7.3	15.8	6.2	10.9	
1174	Slc10a7	Mus musculus solute carrier family 10 (sodium/bile acid cotransporter family), member 7 (Slc10a7), mRNA [NM_029736]	320.1	422.6	388.1	325.3	412.5	
1204	Slc11a2	Mus musculus B16 F10Y cells cDNA, RIKEN full-length enriched library, clone:G370074D17 product:solute carrier family 11 (proton-coupled divalent metal ion transporters), member 2, full insert sequence. [AK148276]	1552.6	1794.5	2951.1	2651.6	2752.6	
1229	Slc29a3	Mus musculus adult male testis cDNA, RIKEN full-length enriched library, clone:4933435C21 product:similar to EQUILIBRATIVE NUCLEOSIDE TRANSPORTER 3 [Mus musculus], full insert sequence. [AK017065]	2.8	2.0	7.8	3.8	2.3	
1304	Slc25a25	Mus musculus solute carrier family 25 (mitochondrial carrier, phosphate carrier), member 25 (Slc25a25), mRNA [NM_146118]	6545.6	8743.8	6759.4	9772.9	3314.9	
1377	Slc6a19	Mus musculus solute carrier family 6 (neurotransmitter transporter), member 19 (Slc6a19), mRNA [NM_028878]	8812.5	11467.2	12583.7	7069.8	9083.7	
1388	Abca14	Mus musculus ATP-binding cassette, sub-family A (ABC1), member 14 (Abca14), mRNA [NM_026458]	−0.6	0.4	0.5	0.2	3.5	
1417	Abca13	Mus musculus ATP-binding cassette, sub-family A (ABC1), member 13 (Abca13), mRNA [NM_178259]	149.3	236.1	247.4	213.1	259.7	
1501	Slc22a6	Mus musculus solute carrier family 22 (organic anion transporter), member 6 (Slc22a6), mRNA [NM_008766]	170544.0	160783.1	183199.2	173234.7	159264.7	
1522	Slc5a5	Mus musculus solute carrier family 5 (sodium iodide symporter), member 5 (Slc5a5), mRNA [NM_053248]	6.7	5.3	7.6	6.6	9.1	
1759	Slc18a1	Mus musculus solute carrier family 18 (vesicular monoamine), member 1 (Slc18a1), mRNA [NM_153054]	8285.9	8777.5	8874.4	8173.5	10247.1	
1826	Slc45a4	Mus musculus mRNA for mKIAA1126 protein [AK173104]	58.2	60.0	64.5	61.9	53.3	
1903	Slc37a2	Mus musculus solute carrier family 37 (glycerol-3-phosphate transporter), member 2 (Slc37a2), mRNA [NM_020258]	25.0	26.1	37.8	21.9	25.3	
1912	Slc36a3	Mus musculus solute carrier family 36 (proton/amino acid symporter), member 3 (Slc36a3), mRNA [NM_172258]	5.2	3.8	5.8	2.9	4.2	
1915	Abcc3	Mus musculus ATP-binding cassette, sub-family C (CFTR/MRP), member 3 (Abcc3), mRNA [NM_029600]	88.9	94.3	144.2	80.0	95.5	
2076	Slc26a3	Mus musculus solute carrier family 26, member 3 (Slc26a3), mRNA [NM_021353]	1.7	−1.3	2.5	2.1	−0.8	
2203	Slc35a5	Mus musculus solute carrier family 35, member A5 (Slc35a5), mRNA [NM_028756]	258.5	334.4	275.6	234.2	362.7	
2273	Slc7a15	Mus musculus adult male small intestine cDNA, RIKEN full-length enriched library, clone:2010001P20 product:hypothetical Permease for amino acids and related compounds, family I containing protein, full insert sequence. [AK008024]	36.0	33.6	61.5	25.2	33.5	
2390	Slc34a1	Mus musculus solute carrier family 34 (sodium phosphate), member 1 (Slc34a1), mRNA [NM_011392]	169129.0	165781.2	198806.7	139708.5	148609.6	
2499	Slco1a1	Mus musculus solute carrier organic anion transporter family, member 1a1 (Slco1a1), mRNA [NM_013797]	7490.2	7919.1	6525.5	7723.9	8942.2	
2523	Abcd2	Mus musculus ATP-binding cassette, sub-family D (ALD), member 2 (Abcd2), mRNA [NM_011994]	0.5	−1.7	2.8	−0.1	1.7	
2568	Slc26a2	Mus musculus solute carrier family 26 (sulfate transporter), member 2 (Slc26a2), mRNA [NM_007885]	195.5	256.3	281.3	232.1	285.2	
2625	Slc9a8	Mus musculus solute carrier family 9 (sodium/hydrogen exchanger), member 8 (Slc9a8), transcript variant 1, mRNA [NM_148929]	7923.0	8768.7	14354.4	12656.9	13127.5	
2698	Slc27a6	Mus musculus 10 days neonate skin cDNA, RIKEN full-length enriched library, clone:4732438L20 product:similar to VERY LONG-CHAIN ACYL-COA SYNTHETASE HOMOLOG 1 [Homo sapiens], full insert sequence [AK028699]	2.5	−0.9	2.9	7.4	0.1	
2740	Slc6a14	Mus musculus solute carrier family 6 (neurotransmitter transporter), member 14 (Slc6a14), mRNA [NM_020049]	−2.0	−2.0	−1.6	0.0	−2.3	
2834	Slc12a6	Mus musculus solute carrier family 12, member 6 (Slc12a6), transcript variant 2, mRNA [NM_133649]	3367.5	4073.4	2977.7	3017.5	3263.1	
2871	Slc39a8	Mus musculus solute carrier family 39 (metal ion transporter), member 8 (Slc39a8), mRNA [NM_026228]	333.9	406.6	383.9	629.1	555.1	
2896	Slc30a7	Mus musculus solute carrier family 30 (zinc transporter), member 7 (Slc30a7), mRNA [NM_023214]	417.0	406.7	445.4	382.9	478.2	
2905	Slc10a4	Mus musculus solute carrier family 10 (sodium/bile acid cotransporter family), member 4 (Slc10a4), mRNA [NM_173403]	11.2	27.3	43.5	23.5	32.9	
2938	Abcc12	Mus musculus ATP-binding cassette, sub-family C (CFTR/MRP), member 12 (Abcc12), mRNA [NM_172912]	2.2	1.2	2.3	2.6	−2.6	
3024	Slc6a18	Mus musculus solute carrier family 6 (neurotransmitter transporter), member 18 (Slc6a18), mRNA [NM_001040692]	8851.6	6903.1	9880.3	9528.8	7882.0	
3204	Slco1a5	Mus musculus solute carrier organic anion transporter family, member 1a5 (Slco1a5), mRNA [NM_130861]	135.7	153.8	148.9	136.2	179.6	
3313	Slc30a5	Mus musculus solute carrier family 30 (zinc transporter), member 5 (Slc30a5), mRNA [NM_022885]	2506.5	2622.4	2482.3	2089.5	2579.0	
3544	Slc6a12	Mus musculus solute carrier family 6 (neurotransmitter transporter, betaine/GABA), member 12 (Slc6a12), mRNA [NM_133661]	17.8	46.4	122.6	57.4	88.6	
3591	Slc44a3	Mus musculus solute carrier family 44, member 3 (Slc44a3), mRNA [NM_145394]	174.2	167.1	206.2	158.2	167.5	
3624	Slc12a8	Mus musculus solute carrier family 12 (potassium/chloride transporters), member 8 (Slc12a8), mRNA [NM_134251]	24.4	30.0	37.1	33.8	38.6	
3639	Slc8a2	Mus musculus solute carrier family 8 (sodium/calcium exchanger), member 2 (Slc8a2), mRNA [NM_148946]	95.9	92.6	144.7	74.3	95.8	
3648	Slc39a11	Mus musculus solute carrier family 39 (metal ion transporter), member 11 (Slc39a11), mRNA [NM_027216]	5188.4	4849.9	6138.3	5792.8	5989.3	
3707	Slc24a1	Mus musculus solute carrier family 24 (sodium/potassium/calcium exchanger), member 1 (Slc24a1), mRNA [NM_144813]	−0.3	−1.0	−3.9	0.1	−2.8	
3795	Slc9a4	Mus musculus solute carrier family 9 (sodium/hydrogen exchanger), member 4 (Slc9a4), mRNA [NM_177084]	8.2	6.8	7.4	8.7	12.1	
3796	Slc1a7	Mus musculus solute carrier family 1 (glutamate transporter), member 7 (Slc1a7), mRNA [NM_146255]	3.1	1.5	4.3	2.8	1.8	
3823	Abca3	Mus musculus ATP-binding cassette, sub-family A (ABC1), member 3 (Abca3), transcript variant 1, mRNA [NM_013855]	173.0	154.5	268.5	160.0	161.1	
3834	Slc25a43	PREDICTED: Mus musculus gene model 8, (NCBI), transcript variant 1 (Gm8), mRNA [XM_110743]	36.5	34.1	32.9	31.7	51.2	
3881	Slc1a3	Mus musculus 4 days neonate male adipose cDNA, RIKEN full-length enriched library, clone:B430312B09 product:unclassifiable, full insert sequence. [AK046688]	−1.7	−1.9	−0.9	0.4	−1.1	
3954	Slc5a9	Mus musculus solute carrier family 5 (sodium/glucose cotransporter), member 9 (Slc5a9), mRNA [NM_145551]	734.6	924.4	906.0	663.3	1010.1	
3961	Slc16a3	Mus musculus solute carrier family 16 (monocarboxylic acid transporters), member 3 (Slc16a3), mRNA [NM_030696]	86.3	99.3	114.8	93.4	103.1	
4124	Slc7a11	Mus musculus solute carrier family 7 (cationic amino acid transporter, y+ system), member 11 (Slc7a11), mRNA [NM_011990]	−2.3	−0.2	0.1	−1.8	−0.3	
4289	Slc7a6	Mus musculus solute carrier family 7 (cationic amino acid transporter, y+ system), member 6 (Slc7a6), mRNA [NM_178798]	30.9	35.1	31.4	26.0	28.5	
4290	Slc25a24	Mus musculus solute carrier family 25 (mitochondrial carrier, phosphate carrier), member 24 (Slc25a24), mRNA [NM_172685]	8.8	6.0	6.4	8.7	9.0	
4291	Slc22a7	Mus musculus solute carrier family 22 (organic anion transporter), member 7 (Slc22a7), mRNA [NM_144856]	16989.2	22399.4	27976.8	41211.6	40347.9	
4306	Abca5	Mus musculus ATP-binding cassette, sub-family A (ABC1), member 5 (Abca5), mRNA [NM_147219]	35.5	52.2	42.1	51.7	65.1	
4327	Slc26a7	Mus musculus solute carrier family 26, member 7 (Slc26a7), mRNA [NM_145947]	2.8	2.1	5.0	7.8	4.8	
4387	Abcb8	Mus musculus 0 day neonate head cDNA, RIKEN full-length enriched library, clone:4833412N02 product:ATP-BINDING CASSETTE, SUB-FAMILY B, MEMBER 8, MITOCHONDRIAL PRECURSOR (MITOCHONDRIAL ATP-BINDING CASSETTE 1) (M-ABC1), full insert sequence. [AK014688]	2.6	0.8	2.6	−0.9	0.3	
4430	Slc9a2	Mus musculus adult male testis cDNA, RIKEN full-length enriched library, clone:4932415O19 product:SODIUM [AK077026]	5222.0	7162.1	7328.5	4907.4	7147.3	
4499	Slc41a3	Mus musculus solute carrier family 41, member 3 (Slc41a3), transcript variant 1, mRNA [NM_027868]	186.7	228.1	301.4	175.5	244.0	
4623	Slc39a12	Mus musculus solute carrier family 39 (zinc transporter), member 12 (Slc39a12), mRNA [NM_001012305]	1.7	0.1	1.9	−0.4	3.4	
4670	Slc35a4	Mus musculus solute carrier family 35, member A4 (Slc35a4), mRNA [NM_026404]	1279.5	1302.8	1740.9	1166.3	1431.7	
4703	Slc16a13	Mus musculus solute carrier family 16 (monocarboxylic acid transporters), member 13 (Slc16a13), mRNA [NM_172371]	1752.5	1870.7	2980.1	2301.7	1839.1	
4730	Slc4a8	Mus musculus solute carrier family 4 (anion exchanger), member 8 (Slc4a8), mRNA [NM_021530]	8.0	10.6	16.9	10.0	17.8	
4873	Slc35b4	Mus musculus solute carrier family 35, member B4 (Slc35b4), mRNA [NM_021435]	2840.5	3563.6	4158.4	2917.5	4054.5	
4882	Slc30a4	Mus musculus solute carrier family 30 (zinc transporter), member 4 (Slc30a4), mRNA [NM_011774]	1085.5	1511.5	1296.0	977.1	1378.0	
4931	Slc2a4	Mus musculus solute carrier family 2 (facilitated glucose transporter), member 4 (Slc2a4), mRNA [NM_009204]	2929.6	3881.4	4324.3	2956.4	4052.6	
5082	Slc15a3	Mus musculus solute carrier family 15, member 3 (Slc15a3), mRNA [NM_023044]	41.3	40.1	60.0	39.6	44.7	
5228	Abcc2	Mus musculus ATP-binding cassette, sub-family C (CFTR/MRP), member 2 (Abcc2), mRNA [NM_013806]	3471.9	3339.8	4172.6	2804.3	3397.0	
5349	Slc39a8	Mus musculus solute carrier family 39 (metal ion transporter), member 8 (Slc39a8), mRNA [NM_026228]	2507.3	2991.5	3666.2	3418.1	3553.4	
5382	Slc9a8	Mus musculus adult male lung cDNA, RIKEN full-length enriched library, clone:1200006P13 product:unclassifiable, full insert sequence. [AK004622]	9.7	6.9	9.5	10.9	11.0	
5457	Slc38a1	Mus musculus solute carrier family 38, member 1 (Slc38a1), mRNA [NM_134086]	177.7	194.9	251.1	175.6	220.6	
5528	Slc13a2	Mus musculus solute carrier family 13 (sodium-dependent dicarboxylate transporter), member 2 (Slc13a2), mRNA [NM_022411]	705.0	641.8	510.4	772.4	591.6	
5534	Slc38a1	Mus musculus 12 days embryo embryonic body between diaphragm region and neck cDNA, RIKEN full-length enriched library, clone:9430020E10 product:hypothetical protein, full insert sequence. [AK162429]	117.1	119.6	179.0	83.2	111.5	
5761	Slc13a3	Mus musculus solute carrier family 13 (sodium-dependent dicarboxylate transporter), member 3 (Slc13a3), mRNA [NM_054055]	25707.8	21966.7	24078.3	20754.4	19287.4	
5795	Slc2a9	Mus musculus solute carrier family 2 (facilitated glucose transporter), member 9 (Slc2a9), transcript variant a, mRNA [NM_001012363]	18.0	33.3	21.8	24.6	31.7	
5804	Abcf3	Mus musculus ATP-binding cassette, sub-family F (GCN20), member 3 (Abcf3), mRNA [NM_013852]	1452.5	1281.7	1588.7	1413.6	1478.3	
5966	Slc13a4	Mus musculus solute carrier family 13 (sodium/sulfate symporters), member 4 (Slc13a4), mRNA [NM_172892]	2081.7	2941.4	2772.3	1939.1	3238.3	
5999	Slc4a10	Mus musculus solute carrier family 4, sodium bicarbonate cotransporter-like, member 10 (Slc4a10), mRNA [NM_033552]	−1.7	−0.6	−3.4	−2.1	−2.2	
6054	Slc37a1	Mus musculus solute carrier family 37 (glycerol-3-phosphate transporter), member 1 (Slc37a1), mRNA [NM_153062]	110.4	156.0	210.8	96.8	153.5	
6069	Slc14a2	Mus musculus solute carrier family 14 (urea transporter), member 2 (Slc14a2), transcript variant 1, mRNA [NM_207651]	−0.1	353.1	477.7	1.7	50.8	
6209	Slc9a7	Mus musculus solute carrier family 9 (sodium/hydrogen exchanger), isoform 7 (Slc9a7), mRNA [NM_177353]	19.6	19.7	28.5	26.8	32.3	
6216	Slc30a1	Mus musculus solute carrier family 30 (zinc transporter), member 1 (Slc30a1), mRNA [NM_009579]	3343.2	4330.2	4342.9	3482.0	4680.4	
6279	Slc22a20	Mus musculus solute carrier family 22 (organic anion transporter), member 20 (Slc22a20), mRNA [NM_198650]	0.6	2.2	2.0	4.1	3.9	
6284	Slc2a3	Mus musculus solute carrier family 2 (facilitated glucose transporter), member 3 (Slc2a3), mRNA [NM_011401]	62.8	47.1	68.0	48.1	58.0	
6321	Abcb8	Mus musculus ATP-binding cassette, sub-family B (MDR/TAP), member 8 (Abcb8), mRNA [NM_029020]	1727.3	1829.5	2305.3	1718.3	2085.8	
6351	Slc6a6	Mus musculus solute carrier family 6 (neurotransmitter transporter, taurine), member 6 (Slc6a6), mRNA [NM_009320]	15390.4	18973.2	23254.9	13661.2	18930.4	

6404	Slc30a7	Mus musculus adult male spinal cord cDNA, RIKEN full-length enriched library, clone:A330067K20 product:hypothetical protein, full insert sequence [AK039587]	0.0	−1.9	−0.9	−1.1	1.1
6442	Slc28a3	Mus musculus solute carrier family 28 (sodium-coupled nucleoside transporter), member 3 (Slc28a3), mRNA [NM_022317]	0.7	3.3	2.3	4.3	0.8
6479	Slc22a17	Mus musculus solute carrier family 22 (organic cation transporter), member 17 (Slc22a17), mRNA [NM_021551]	574.9	606.5	767.5	586.7	626.9
6533	Slc6a8	Mus musculus solute carrier family 6 (neurotransmitter transporter, creatine), member 8 (Slc6a8), mRNA [NM_133987]	13820.9	17853.9	21777.6	14816.7	16081.4
6622	Slc17a2	Mus musculus solute carrier family 17 (sodium phosphate), member 2 (Slc17a2), mRNA [NM_144836]	36.1	34.5	56.5	27.5	34.4
6693	Slc36a2	Mus musculus 15 days embryo head cDNA, RIKEN full-length enriched library, clone:D930024H10 product:weakly similar to LYSOSOMAL AMINO ACID TRANSPORTER 1 [Rattus norvegicus], full insert sequence. [AK086373]	−2.3	−0.8	−0.1	−0.4	6.7
6708	Slc6a4	Mus musculus solute carrier family 6 (neurotransmitter transporter, serotonin), member 4 (Slc6a4), mRNA [NM_010484]	226.0	335.3	328.5	253.5	333.5
6835	Slc6a14	Mus musculus solute carrier family 6 (neurotransmitter transporter), member 14 (Slc6a14), mRNA [NM_020049]	70.7	83.5	80.8	41.4	69.2
6873	Abcd1	Mus musculus ATP-binding cassette, sub-family D (ALD), member 1 (Abcd1), mRNA [NM_007435]	68.1	85.9	114.6	58.4	74.0
6983	Slc27a2	Mus musculus fatty acid transport protein 2 mRNA, complete cds. [AF072757]	19151.8	20947.0	14703.1	23545.2	24508.5
7051	Slc8a1	Mus musculus solute carrier family 8 (sodium/calcium exchanger), member 1 (Slc8a1), mRNA [NM_011406]	61.8	71.0	61.8	70.2	88.7
7060	Slc25a26	Mus musculus solute carrier family 25 (mitochondrial carrier, phosphate carrier), member 26 (Slc25a26), mRNA [NM_026255]	1226.9	1272.1	1549.0	1221.7	1422.1
7113	Slc5a3	Mus musculus solute carrier family 5 (inositol transporters), member 3 (Slc5a3), mRNA [NM_017391]	141.4	160.3	169.8	107.6	122.4
7133	Slc22a8	Mus musculus solute carrier family 22 (organic anion transporter), member 8 (Slc22a8), mRNA [NM_031194]	433.2	552.2	533.9	383.1	367.3
7157	Slc4a7	Mus musculus solute carrier family 4, sodium bicarbonate cotransporter, member 7 (Slc4a7), mRNA [NM_001033270]	358.6	416.4	396.2	318.4	420.1
7430	Slc8a3	Mus musculus solute carrier family 8 (sodium/calcium exchanger), member 3 (Slc8a3), mRNA [NM_080440]	−3.1	−0.7	−2.1	−0.5	−0.4
7444	Slc2a4	Mus musculus solute carrier family 2 (facilitated glucose transporter), member 4 (Slc2a4), mRNA [NM_009204]	2486.9	3259.6	3900.3	2819.6	3511.2
7460	Slc43a3	Mus musculus solute carrier family 43, member 3 (Slc43a3), mRNA [NM_021398]	1683.7	1780.8	1582.4	1201.7	1432.1
7594	Slc24a4	Mus musculus solute carrier family 24 (sodium/potassium/calcium exchanger), member 4 (Slc24a4), mRNA [NM_172152]	80.3	106.0	143.5	91.7	120.3
7607	Slco6b1	Mus musculus solute carrier organic anion transporter family, member 6b1 (Slco6b1), mRNA [NM_001039475]	−1.8	−1.0	−4.4	−3.6	−2.9
7697	Abcc9	Mus musculus ATP-binding cassette, sub-family C (CFTR/MRP), member 9 (Abcc9), transcript variant 2, mRNA [NM_021041]	71.2	88.4	96.9	68.6	76.8
7717	Abcf2	Mus musculus ATP-binding cassette, sub-family F (GCN20), member 2 (Abcf2), mRNA [NM_013853]	1541.5	1520.3	2171.3	1569.9	1834.8
7779	Slc1a2	Mus musculus adult male testis cDNA, RIKEN full-length enriched library, clone:1700091C19 product:inferred: Mus musculus adult male testis cDNA, RIKEN full-length enriched library, clone:4933426I16, full insert sequence, full insert sequence. [AK018918]	160.1	121.3	143.8	137.7	91.0
7796	Slc2a1	Mus musculus solute carrier family 2 (facilitated glucose transporter), member 1 (Slc2a1), mRNA [NM_011400]	2955.5	4389.7	5663.2	3442.4	5077.8
7821	Slc27a1	Mus musculus solute carrier family 27 (fatty acid transporter), member 1 (Slc27a1), mRNA [NM_011977]	597.7	622.8	706.7	572.0	589.5
8046	Slc10a4	Mus musculus solute carrier family 10 (sodium/bile acid cotransporter family), member 4 (Slc10a4), mRNA [NM_173403]	0.3	1.4	−1.0	0.8	0.3
8154	Slc38a2	Mus musculus 13 days embryo heart cDNA, RIKEN full-length enriched library, clone:D330050A21 product:solute carrier family 38, member 2, full insert sequence [AK164568]	250.0	228.8	320.0	259.2	225.6
8157	Slc22a5	Mus musculus solute carrier family 22 (organic cation transporter), member 5 (Slc22a5), mRNA [NM_011396]	8784.0	10174.3	10005.8	8841.6	8637.8
8242	Slc38a1	Mus musculus 12 days embryo embryonic body between diaphragm region and neck cDNA, RIKEN full-length enriched library, clone:9430020E10 product:hypothetical protein, full insert sequence. [AK162429]	16.6	22.1	21.9	18.7	19.1
8266	Slco5a1	Mus musculus 10 days neonate skin cDNA, RIKEN full-length enriched library, clone:4732454B15 product:similar to ORGANIC ANION TRANSPORTER POLYPEPTIDE-RELATED PROTEIN 4 [Homo sapiens], full insert sequence. [AK028758]	0.2	−0.5	−2.6	1.2	−0.1
8269	Slc9a3r1	Mus musculus solute carrier family 9 (sodium/hydrogen exchanger), isoform 3 regulator 1 (Slc9a3r1), mRNA [NM_012030]	10540.7	11181.0	13527.7	8423.3	11918.9
8325	Slc35a5	Mus musculus solute carrier family 35, member A5 (Slc35a5), mRNA [NM_028756]	73.6	83.3	94.0	67.3	75.0
8457	Slc2a2	Mus musculus solute carrier family 2 (facilitated glucose transporter), member 2 (Slc2a2), mRNA [NM_031197]	2926.7	2626.5	2576.5	2576.1	2913.5
8487	Slc22a21	Mus musculus solute carrier family 22 (organic cation transporter), member 21 (Slc22a21), mRNA [NM_019723]	1982.8	2063.7	1968.9	2109.5	1888.9
8509	Abca3	Mus musculus B6-derived CD11 +ve dendritic cells cDNA, RIKEN full-length enriched library, clone:F730004H09 product:ATP-binding cassette, sub-family A (ABC1), member 3, full insert sequence. [AK089316]	4.2	1.4	−3.3	−1.0	0.9
8666	Slc2a2	Mus musculus solute carrier family 2 (facilitated glucose transporter), member 2 (Slc2a2), mRNA [NM_031197]	11408.0	12073.6	13060.5	8681.9	10787.7
8817	Slc6a11	Mus musculus solute carrier family 6 (neurotransmitter transporter, GABA), member 11 (Slc6a11), mRNA [NM_172890]	17.5	24.1	33.3	18.3	24.6
8827	Slc27a2	Mus musculus solute carrier family 27 (fatty acid transporter), member 2 (Slc27a2), mRNA [NM_011978]	143023.5	154502.3	172887.1	141655.0	144195.7
8873	Slc29a3	Mus musculus solute carrier family 29 (nucleoside transporters), member 3 (Slc29a3), mRNA [NM_023596]	76.4	81.7	102.7	63.6	79.2
8962	Slc25a27	Mus musculus solute carrier family 25, member 27 (Slc25a27), mRNA [NM_028711]	71.2	83.6	97.6	67.6	70.0
9086	Slc35f1	Mus musculus solute carrier family 35, member F1 (Slc35f1), mRNA [NM_178675]	199.1	239.3	255.5	241.8	262.4
9158	Slc38a1	Mus musculus solute carrier family 38, member 1 (Slc38a1), mRNA [NM_134086]	3.1	8.0	4.1	5.5	4.1
9175	Abcd1	Mus musculus ATP-binding cassette protein (Abcd1) mRNA, partial cds. [AF213385]	44.7	56.2	60.5	40.7	48.7
9197	Slc35a2	Mus musculus solute carrier family 35 (UDP-galactose transporter), member 2 (Slc35a2), mRNA [NM_078484]	317.8	323.6	404.0	318.6	350.5
9234	Abcd2	Mus musculus ATP-binding cassette, sub-family D (ALD), member 2 (Abcd2), mRNA [NM_011994]	−0.7	−0.4	0.5	1.8	1.5
9236	Slc1a2	Mus musculus solute carrier family 1 (glial high affinity glutamate transporter), member 2 (Slc1a2), transcript variant 3, mRNA [NM_011393]	32.8	43.4	47.4	34.2	35.5
9284	Slc10a2	Mus musculus solute carrier family 10, member 2 (Slc10a2), mRNA [NM_011388]	1105.3	948.5	1285.7	928.3	1279.5
9343	Slc2a13	Mus musculus 3 days neonate thymus cDNA, RIKEN full-length enriched library, clone:A630029G22 product:PROTON MYO-INOSITOL TRANSPORTER homolog [Rattus norvegicus], full insert sequence. [AK041673]	−4.2	−3.2	−1.6	0.7	−0.7
9466	Slc1a3	Mus musculus solute carrier family 1 (glial high affinity glutamate transporter), member 3 (Slc1a3), mRNA [NM_148938]	54.8	86.0	87.7	67.5	87.7
9584	Abca6	Mus musculus ATP-binding cassette, sub-family A (ABC1), member 6 (Abca6), mRNA [NM_147218]	−0.2	1.6	1.2	−1.6	−0.9
9587	Slc25a26	Mus musculus 16 days embryo head cDNA, RIKEN full-length enriched library, clone:C130049G05 product:unclassifiable, full insert sequence [AK048325]	2.5	−0.9	0.4	0.8	1.4
9592	Slc16a14	Mus musculus solute carrier family 16 (monocarboxylic acid transporters), member 14 (Slc16a14), mRNA [NM_027921]	304.7	239.0	221.7	219.3	250.6
9612	Slc4a4	Mus musculus adult male medulla oblongata cDNA, RIKEN full-length enriched library, clone:6330501O17 product:unclassifiable, full insert sequence. [AK134619]	2813.1	3565.2	2567.6	2436.4	2641.9
9646	Slc2a9	Mus musculus solute carrier family 2 (facilitated glucose transporter), member 9 (Slc2a9), transcript variant b, mRNA [NM_145559]	219.9	313.5	203.3	149.2	251.2
9747	Slc33a1	Mus musculus solute carrier family 33 (acetyl-CoA transporter), member 1 (Slc33a1), mRNA [NM_015728]	163.5	216.7	155.2	228.5	326.1
9778	Slc25a13	Mus musculus solute carrier family 25 (mitochondrial carrier, adenine nucleotide translocator), member 13 (Slc25a13), mRNA [NM_015829]	9631.4	12206.9	11640.5	9071.7	10876.2
9818	Slc6a7	Mus musculus solute carrier family 6 (neurotransmitter transporter, L-proline), member 7 (Slc6a7), mRNA [NM_201353]	13.9	17.5	18.2	14.4	18.4
9846	Slc39a1	Mus musculus solute carrier family 39 (zinc transporter), member 1 (Slc39a1), mRNA [NM_013901]	2297.1	2543.7	3061.5	1915.3	2530.8
9847	Slc39a7	Mus musculus solute carrier family 39 (zinc transporter), member 7 (Slc39a7), transcript variant 1, mRNA [NM_008202]	21178.1	21191.7	26596.5	23087.7	24474.2
9916	Slc16a2	Mus musculus solute carrier family 16 (monocarboxylic acid transporters), member 2 (Slc16a2), mRNA [NM_009197]	24061.8	24351.1	30877.7	20362.7	22792.6
9946	Slc5a1	Mus musculus solute carrier family 5 (sodium/glucose cotransporter), member 1 (Slc5a1), mRNA [NM_019810]	796.9	672.9	842.1	868.0	940.3
9990	Slc2a8	Mus musculus solute carrier family 2, (facilitated glucose transporter), member 8 (Slc2a8), mRNA [NM_019488]	729.9	855.3	1085.3	628.8	837.6
10012	Abcc1	Mus musculus ATP-binding cassette, sub-family C (CFTR/MRP), member 1 (Abcc1), mRNA [NM_008576]	159.1	231.1	315.0	237.9	286.4
10045	Slc12a1	Mus musculus apical bumetanide-sensitive Na-(K)-Cl cotransporter alternatively spliced isoform mBSC1-A4 (Slc12a1) mRNA, complete cds. [U61381]	15.9	25.3	27.9	25.5	20.5
10082	Abcf1	Mus musculus ATP-binding cassette, sub-family F (GCN20), member 1 (Abcf1), mRNA [NM_013854]	6699.9	8656.7	9174.1	7698.7	8731.1
10199	Slc15a2	Mus musculus solute carrier family 15 (H+/peptide transporter), member 2 (Slc15a2), mRNA [NM_021301]	2779.6	2652.2	2564.9	1973.7	2389.7
10330	Slc15a1	Mus musculus solute carrier family 15 (oligopeptide transporter), member 1 (Slc15a1), mRNA [NM_053079]	0.3	3.0	0.1	1.2	1.9
10343	Slc4a3	Mus musculus 0 day neonate head cDNA, RIKEN full-length enriched library, clone:4833445E16 product:hypothetical protein, full insert sequence. [AK161240]	21.4	18.9	25.2	21.1	16.4
10373	Slc22a8	Mus musculus solute carrier family 22 (organic anion transporter), member 8 (Slc22a8), mRNA [NM_031194]	2758.5	3037.4	3508.5	1533.4	1743.1
10392	Abcb1a	Mus musculus ATP-binding cassette, sub-family B (MDR/TAP), member 1A (Abcb1a), mRNA [NM_011076]	36.8	63.6	33.9	34.0	39.2
10406	Slc37a4	Mus musculus solute carrier family 37 (glycerol-6-phosphate transporter), member 4 (Slc37a4), mRNA [NM_008063]	80939.2	82793.2	78355.2	60830.5	72281.0
10474	Slc2a4	Mus musculus solute carrier family 2 (facilitated glucose transporter), member 4 (Slc2a4), mRNA [NM_009204]	2913.1	3739.8	4161.2	3088.0	3865.6
10580	Slc27a2	Mus musculus adult male hypothalamus cDNA, RIKEN full-length enriched library, clone:A230068F22 product:solute carrier family 27 (fatty acid transporter), member 2, full insert sequence. [AK038847]	304.0	260.5	262.1	199.1	256.0
10773	Slc12a2	Mus musculus solute carrier family 12, member 2 (Slc12a2), mRNA [NM_009194]	1594.6	1950.3	2256.2	1776.0	2149.8
10875	Abcc3	Mus musculus ATP-binding cassette, sub-family C (CFTR/MRP), member 3 (Abcc3), mRNA [NM_029600]	26.3	0.5	5.0	1.9	2.4
11193	Slc5a4a	Mus musculus solute carrier family 5, member 4a (Slc5a4a), mRNA [NM_133184]	−0.6	0.9	0.0	1.0	−0.2
11237	Slc17a3	Mus musculus solute carrier family 17 (sodium phosphate), member 3 (Slc17a3), mRNA [NM_134069]	75889.7	77167.2	89610.9	67947.7	86698.4
11337	Slc16a8	Mus musculus solute carrier family 16 (monocarboxylic acid transporters), member 8 (Slc16a8), mRNA [NM_020516]	61.5	69.0	99.7	63.6	101.0
11368	Slc25a17	Mus musculus solute carrier family 25 (mitochondrial carrier, peroxisomal membrane protein), member 17 (Slc25a17), mRNA [NM_011399]	1169.8	1876.4	1729.9	1141.3	1698.2
11384	Slc25a18	PREDICTED: Mus musculus solute carrier family 25 (mitochondrial carrier), member 18 (Slc25a18), mRNA [XM_110620]	12.5	12.1	20.6	11.5	14.8
11390	Slco6d1	Mus musculus adult male testis cDNA, RIKEN full-length enriched library, clone:4921511I05 product:solute carrier organic anion transporter family, member 6d1, full insert sequence. [AK014872]	8.0	0.3	−1.5	0.0	0.7
11498	Slc9a2	Mus musculus solute carrier family 9 (sodium/hydrogen exchanger), member 2 (Slc9a2), mRNA [NM_001033289]	62.5	74.2	72.2	61.9	69.5
11580	Slc7a6os	Mus musculus solute carrier family 7, member 6 opposite strand (Slc7a6os), mRNA [NM_001007567]	1320.9	1446.6	1683.7	1131.5	1395.5
11789	Slc25a4	Mus musculus solute carrier family 25 (mitochondrial carrier, adenine nucleotide translocator), member 4 (Slc25a4), mRNA [NM_007450]	33317.5	36608.3	46432.1	29470.2	37413.1
11818	Slc36a4	Mus musculus solute carrier family 36 (proton/amino acid symporter), member 4 (Slc36a4), mRNA [NM_172289]	23.7	36.0	40.0	36.4	57.9
11829	Slc25a2	Mus musculus adult male testis cDNA, RIKEN full-length enriched library, clone:4933415J19 product:ornithine transporter 2, full insert sequence. [AK077159]	66.3	41.6	42.5	57.0	41.0
12052	Slc25a32	Mus musculus solute carrier family 25, member 32 (Slc25a32), mRNA [NM_172402]	554.8	757.8	755.9	490.9	740.7
12101	Slc25a10	Mus musculus solute carrier family 25 (mitochondrial carrier, dicarboxylate transporter), member 10 (Slc25a10), mRNA [NM_013770]	48560.5	60225.4	52047.2	47320.4	52761.9
12113	Slc31a1	Mus musculus solute carrier family 31, member 1 (Slc31a1), mRNA [NM_175090]	2615.8	3166.1	2845.4	2754.8	2836.2
12149	Slc25a14	Mus musculus 12 days embryo spinal ganglion cDNA, RIKEN full-length enriched library, clone:D130006J18 product:solute carrier family 25 (mitochondrial carrier, brain), member 14, full insert sequence. [AK051145]	5.1	4.2	2.8	7.4	4.1
12202	Slc7a7	Mus musculus solute carrier family 7 (cationic amino acid transporter, y+ system), member 7 (Slc7a7), mRNA [NM_011405]	84200.2	91139.2	112981.6	64971.1	76562.9
12204	Slc36a3	Mus musculus solute carrier family 36 (proton/amino acid symporter), member 3 (Slc36a3), mRNA [NM_172258]	9.0	6.1	7.8	4.4	8.0
12240	Abcb9	Mus musculus ATP-binding cassette, sub-family B (MDR/TAP), member 9 (Abcb9), mRNA [NM_019875]	2666.6	3333.6	3579.0	2275.1	3532.5
12267	Slc30a6	Mus musculus solute carrier family 30 (zinc transporter), member 6 (Slc30a6), mRNA [NM_144798]	482.7	453.5	563.6	351.9	432.9
12420	Slc38a4	Mus musculus solute carrier family 38, member 4 (Slc38a4), mRNA [NM_027052]	76.4	78.3	84.0	69.1	91.9
12525	Slc31a2	Mus musculus solute carrier family 31, member 2 (Slc31a2), mRNA [NM_025286]	1232.1	1359.3	1361.9	1021.0	1221.0
12548	Slc3a1	Mus musculus solute carrier family 3, member 1 (Slc3a1), mRNA [NM_009205]	47386.2	46179.4	56254.1	39317.3	41156.9
12623	Slc25a20	Mus musculus solute carrier family 25 (mitochondrial carnitine/acylcarnitine translocase), member 20 (Slc25a20), mRNA [NM_020520]	2283.4	2220.5	2184.0	1927.8	1897.7
12683	Slc35e4	Mus musculus solute carrier family 35, member E4 (Slc35e4), mRNA [NM_153142]	663.6	695.0	820.0	783.9	811.5
12690	Slc17a8	Mus musculus solute carrier family 17 (sodium-dependent inorganic phosphate cotransporter), member 8 (Slc17a8), mRNA [NM_182959]	3.6	7.7	6.8	6.3	6.6

12813	Slc14a2	Mus musculus solute carrier family 14 (urea transporter), member 2 (Slc14a2), transcript variant 2, mRNA [NM_030683]	111.1	104.0	148.7	80.9	114.6
12925	Slc35c2	Mus musculus solute carrier family 35, member C2 (Slc35c2), mRNA [NM_144893]	1249.8	1307.0	1950.7	1248.7	1374.7
12965	Slc24a4	Mus musculus adult retina cDNA, RIKEN full-length enriched library, clone:A930002M03 product:weakly similar to SODIUM/POTASSIUM/CALCIUM EXCHANGER 3 PRECURSOR (NA(+)/K(+)/CA(2+)-EXCHANGE PROTEIN 3) [Mus musculus], full insert sequence. [AK044239]	1.8	0.5	−0.9	−2.1	−1.0
13004	Slc43a1	Mus musculus solute carrier family 43, member 1, mRNA (cDNA clone MGC:59491 IMAGE:6330614), complete cds. [BC053747]	32.7	32.8	39.1	41.8	35.5
13178	Slc25a30	Mus musculus solute carrier family 25, member 30 (Slc25a30), mRNA [NM_026232]	1559.7	1454.7	1917.8	2118.7	1751.7
13222	Slc5a10	Mus musculus solute carrier family 5 (sodium/glucose cotransporter), member 10 (Slc5a10), mRNA [NM_001033227]	33449.4	36851.6	45951.5	27964.3	28434.0
13287	Slc12a1	Mus musculus solute carrier family 12, member 1 (Slc12a1), transcript variant 3, mRNA [NM_001079690]	14563.1	24919.0	31729.2	20209.9	23524.7
13364	Slc30a10	Mus musculus solute carrier family 30, member 10 (Slc30a10), mRNA [NM_001033286]	−2.6	−2.7	−2.3	−1.8	−2.4
13472	Abcc5	Mus musculus ATP-binding cassette, sub-family C (CFTR/MRP), member 5 (Abcc5), transcript variant 2, mRNA [NM_176839]	282.6	276.0	279.1	267.3	252.3
13506	Slc5a2	Mus musculus solute carrier family 5 (sodium/glucose cotransporter), member 2 (Slc5a2), mRNA [NM_133254]	55637.1	45355.4	67796.8	44765.5	52083.9
13724	Slc9a1	Mus musculus Na+/H+ exchanger (NHE-1) mRNA, complete cds. [U51112]	275.7	247.1	330.8	188.2	218.3
13767	Slc7a11	Mus musculus solute carrier family 7 (cationic amino acid transporter, y+ system), member 11 (Slc7a11), mRNA [NM_011990]	−0.3	0.4	−0.3	0.8	−1.0
13852	Slc29a2	Mus musculus solute carrier family 29 (nucleoside transporters), member 2 (Slc29a2), mRNA [NM_007854]	156.8	174.5	240.2	144.0	164.7
13873	Slc4a1	Mus musculus solute carrier family 4 (anion exchanger), member 1 (Slc4a1), mRNA [NM_011403]	1467.3	1921.4	2913.9	1484.2	2080.6
13874	Slc10a3	Mus musculus solute carrier family 10 (sodium/bile acid cotransporter family), member 3 (Slc10a3), mRNA [NM_145406]	752.1	960.8	1308.4	747.4	1069.9
13946	Slc37a2	Mus musculus solute carrier family 37 (glycerol-3-phosphate transporter), member 2 (Slc37a2), mRNA [NM_020258]	22.7	22.2	29.3	21.6	20.4
14060	Slc28a3	Mus musculus solute carrier family 28 (sodium-coupled nucleoside transporter), member 3 (Slc28a3), mRNA [NM_022317]	47.9	45.4	52.6	39.4	39.7
14250	Abca7	Mus musculus ATP-binding cassette, sub-family A (ABC1), member 7 (Abca7), mRNA [NM_013850]	2.5	0.6	4.5	3.2	2.7
14278	Slc7a1	Mus musculus solute carrier family 7 (cationic amino acid transporter, y+ system), member 1 (Slc7a1), mRNA [NM_007513]	27.5	27.2	31.4	21.6	24.3
14623	Slc25a46	Mus musculus solute carrier family 25, member 46 (Slc25a46), mRNA [NM_026165]	30.4	42.1	53.6	30.2	47.9
14625	Slc39a2	Mus musculus solute carrier family 39 (zinc transporter), member 2 (Slc39a2), mRNA [NM_001039676]	47.3	36.5	74.7	28.9	30.4
14727	Slc2a6	Mus musculus solute carrier family 2 (facilitated glucose transporter), member 6 (Slc2a6), mRNA [NM_172659]	47.0	48.1	61.5	42.6	59.0
14833	Slc10a2	Mus musculus solute carrier family 10, member 2 (Slc10a2), mRNA [NM_011388]	629.2	625.2	806.8	742.5	748.0
15168	Slc25a23	Mus musculus solute carrier family 25 (mitochondrial carrier; phosphate carrier), member 23 (Slc25a23), mRNA [NM_025877]	114.6	120.9	194.6	66.2	94.7
15206	Slc22a18	Mus musculus solute carrier family 22 (organic cation transporter), member 18 (Slc22a18), transcript variant 2, mRNA [NM_001042760]	6479.5	5641.2	8039.7	4995.4	5889.6
15292	Abca8b	Mus musculus ATP-binding cassette, sub-family A (ABC1), member 8b (Abca8b), mRNA [NM_013851]	4.5	1.8	4.7	0.7	2.8
15298	Slc38a3	Mus musculus solute carrier family 38, member 3 (Slc38a3), mRNA [NM_023805]	552.8	664.7	1277.9	703.6	832.7
15360	Slc17a8	Mus musculus solute carrier family 17 (sodium-dependent inorganic phosphate cotransporter), member 8 (Slc17a8), mRNA [NM_182959]	65.6	67.2	85.4	64.6	66.6
15406	Slc11a2	Mus musculus solute carrier family 11 (proton-coupled divalent metal ion transporters), member 2 (Slc11a2), mRNA [NM_008732]	2769.9	2985.5	2834.4	2053.5	3011.5
15427	Slc30a9	Mus musculus adult male diencephalon cDNA, RIKEN full-length enriched library, clone:9330134J17 product:HUEL-LIKE PROTEIN (FRAGMENT) homolog [Mus musculus], full insert sequence [AK033990]	578.6	657.4	732.5	619.5	621.4
15508	Slc43a2	Mus musculus 1 month neonate cerebellum cDNA, RIKEN full-length enriched library, clone:G630018E19 product:similar to PB39 (PROSTATE CANCER OVEREXPRESSED GENE 1) [Homo sapiens], full insert sequence. [AK090207]	751.4	1055.3	1222.4	1588.8	919.4
15728	Slc5a4b	Mus musculus solute carrier family 5 (neutral amino acid transporters, system A), member 4b (Slc5a4b), mRNA [NM_023219]	21.5	17.2	29.8	18.8	26.0
15767	Slc39a5	Mus musculus solute carrier family 39 (metal ion transporter), member 5 (Slc39a5), mRNA [NM_028051]	739.1	582.3	772.2	751.1	614.4
16018	Slc25a37	Mus musculus solute carrier family 25, member 37 (Slc25a37), mRNA [NM_026331]	95.5	84.3	105.3	88.0	95.6
16143	Abca15	Mus musculus ATP-binding cassette, sub-family A (ABC1), member 15 (Abca15), mRNA [NM_177213]	−0.5	−0.5	−1.3	−0.1	−1.6
16399	Slc30a8	Mus musculus solute carrier family 30 (zinc transporter), member 8 (Slc30a8), mRNA [NM_172816]	6.7	4.7	4.1	3.1	2.4
16421	Slc28a2	Mus musculus solute carrier family 28 (sodium-coupled nucleoside transporter), member 2 (Slc28a2), mRNA [NM_172980]	13.3	13.9	16.9	8.0	14.0
16594	Slc5a7	Mus musculus solute carrier family 5 (choline transporter), member 7 (Slc5a7), mRNA [NM_022025]	−2.7	−5.4	−4.4	−3.9	−3.1
16664	Slc8a1	Mus musculus sodium-calcium exchanger (NCX1) mRNA, complete cds. [AF004666]	776.4	711.9	807.8	689.3	1035.3
16752	Slc36a2	Mus musculus solute carrier family 36 (proton/amino acid symporter), member 2 (Slc36a2), mRNA [NM_153170]	9.6	8.3	11.4	5.0	5.0
16757	Slc31a2	Mus musculus solute carrier family 31, member 2 (Slc31a2), mRNA [NM_025286]	397.6	418.8	505.5	378.1	480.1
16761	Slc5a12	Mus musculus adult male kidney cDNA, RIKEN full-length enriched library, clone:F530008H01 product:hypothetical Sodium:solute symporter family profile containing protein, full insert sequence. [AK143967]	45.6	50.6	48.4	33.8	32.7
16884	Slc18a2	Mus musculus solute carrier family 18 (vesicular monoamine), member 2 (Slc18a2), mRNA [NM_172523]	45.5	45.4	48.6	29.9	30.1
16894	Slc9a3	solute carrier family 9 (sodium/hydrogen exchanger), member 3 [Source:MarkerSymbol;Acc:MGI:105064] [ENSMUST00000036208]	70.6	74.1	79.3	53.0	62.4
16906	Slc25a39	Mus musculus solute carrier family 25, member 39 (Slc25a39), mRNA [NM_026542]	3696.2	3159.3	3981.7	3100.3	3400.9
17013	Slc24a2	Mus musculus solute carrier family 24 (sodium/potassium/calcium exchanger), member 2 (Slc24a2), mRNA [NM_172426]	1.0	−0.8	−0.4	0.3	−0.6
17187	Slc25a14	Mus musculus solute carrier family 25 (mitochondrial carrier, brain), member 14 (Slc25a14), mRNA [NM_011398]	1884.7	2069.1	2434.8	1413.2	2202.3
17268	Slc12a7	Mus musculus solute carrier family 12, member 7 (Slc12a7), mRNA [NM_011390]	10155.6	11075.2	13886.3	10534.5	11276.5
17369	Slc30a5	Mus musculus 12 days embryo spinal ganglion cDNA, RIKEN full-length enriched library, clone:D130062D18 product:unclassifiable, full insert sequence. [AK051654]	80.0	53.2	57.9	44.9	57.6
17465	Slc35a3	Mus musculus solute carrier family 35 (UDP-N-acetylglucosamine (UDP-GlcNAc) transporter), member 3 (Slc35a3), mRNA [NM_144902]	4020.4	4268.2	5843.3	3721.3	5192.4
17473	Slc25a21	Mus musculus 9.5 days embryo parthenogenote cDNA, RIKEN full-length enriched library, clone:B130014P16 product:MOTOCHONDRIAL OXODICARBOXYLATE CARRIER homolog [Rattus norvegicus], full insert sequence. [AK044945]	−1.5	−1.4	−0.3	2.2	−2.4
17533	Slc6a1	Mus musculus solute carrier family 6 (neurotransmitter transporter, GABA), member 1 (Slc6a1), mRNA [NM_178703]	3.2	2.1	3.8	3.8	3.9
17601	Slc25a46	Mus musculus solute carrier family 25, member 46 (Slc25a46), mRNA [NM_026165]	508.6	492.1	533.5	540.8	617.1
17703	Slc14a1	Mus musculus solute carrier family 14 (urea transporter), member 1 (Slc14a1), mRNA [NM_028122]	4.8	57.9	190.1	76.2	169.1
17707	Slc35f1	Mus musculus solute carrier family 35, member F1 (Slc35f1), mRNA [NM_178675]	217.1	205.7	211.0	356.9	242.2
17923	Slc1a6	Mus musculus solute carrier family 1 (high affinity aspartate/glutamate transporter), member 6 (Slc1a6), mRNA [NM_009200]	27.9	29.4	38.8	27.2	30.0
17942	Slc4a10	Mus musculus solute carrier family 4, sodium bicarbonate cotransporter-like, member 10 (Slc4a10), mRNA [NM_033552]	1.4	−0.6	−1.4	2.3	1.4
18088	Abca13	Mus musculus ATP-binding cassette, sub-family A (ABC1), member 13 (Abca13), mRNA [NM_178259]	138.3	166.4	163.4	203.3	183.1
18171	Slc35d1	Mus musculus 16 days neonate thymus cDNA, RIKEN full-length enriched library, clone:A130065C03 product:hypothetical protein, full insert sequence [AK037934]	84.3	62.2	63.2	88.2	54.5
18179	Slc8a1	Mus musculus sodium/calcium exchanger isoform NaCa3 (Ncx1) mRNA, partial cds. [AF108396]	129.2	107.1	140.3	115.7	159.1
18437	Slc7a10	Mus musculus solute carrier family 7 (cationic amino acid transporter, y+ system), member 10 (Slc7a10), mRNA [NM_017394]	−0.7	−3.9	5.5	3.7	1.7
18592	Slco2a1	Mus musculus prostaglandin transporter PGT mRNA, complete cds. [AF323958]	239.2	252.3	330.2	251.9	255.9
18687	Slc2a9	Mus musculus solute carrier family 2 (facilitated glucose transporter), member 9 (Slc2a9), transcript variant a, mRNA [NM_001012363]	168.5	193.3	181.2	152.1	189.6
18773	Abcg2	Mus musculus ATP-binding cassette, sub-family G (WHITE), member 2 (Abcg2), mRNA [NM_011920]	64747.6	65034.9	77912.3	55848.3	58086.2
18810	Slc1a1	Mus musculus solute carrier family 1 (neuronal/epithelial high affinity glutamate transporter, system Xag), member 1 (Slc1a1), mRNA [NM_009199]	6713.5	7495.3	9129.7	5781.9	8072.0
18811	Abcc8	Mus musculus ATP-binding cassette, sub-family C (CFTR/MRP), member 8 (Abcc8), mRNA [NM_011510]	18.7	17.8	37.0	10.6	18.8
18829	Slc25a27	Mus musculus solute carrier family 25, member 27 (Slc25a27), mRNA [NM_028711]	10.6	9.2	12.8	8.7	7.6
19028	Slc8a1	Mus musculus solute carrier family 8 (sodium/calcium exchanger), member 1 (Slc8a1), mRNA [NM_011406]	109.2	149.0	187.3	163.3	250.5
19053	Slc4a4	Mus musculus solute carrier family 4 (anion exchanger), member 4 (Slc4a4), mRNA [NM_018760]	1922.4	2115.2	2534.4	2332.4	2399.7
19224	Slc5a6	Mus musculus solute carrier family 5 (sodium-dependent vitamin transporter), member 6 (Slc5a6), mRNA [NM_177870]	2002.7	2686.6	2319.5	1765.4	2444.4
19232	Slc2a13	Mus musculus solute carrier family 2 (facilitated glucose transporter), member 13 (Slc2a13), mRNA [NM_001033633]	195.5	450.3	494.3	283.0	400.8
19334	Slc2a4	Mus musculus solute carrier family 2 (facilitated glucose transporter), member 4 (Slc2a4), mRNA [NM_009204]	3530.9	4191.5	5036.5	3679.1	4916.8
19380	Slc25a35	Mus musculus adult male urinary bladder cDNA, RIKEN full-length enriched library, clone:9530097O21 product:RAN guanine nucleotide release factor, full insert sequence. [AK035739]	978.5	414.2	453.0	296.9	434.4
19399	Slc25a25	Mus musculus solute carrier family 25 (mitochondrial carrier, phosphate carrier), member 25 (Slc25a25), mRNA [NM_146118]	12697.5	15374.6	12827.1	16762.3	5242.3
19560	Slc7a11	Mus musculus NOD-derived CD11c +ve dendritic cells cDNA, RIKEN full-length enriched library, clone:F630119J15 product:solute carrier family 7 (cationic amino acid transporter, y+ system), member 11, full insert sequence. [AK089283]	0.0	−2.0	10.2	1.5	−0.6
19635	Slc35f5	Mus musculus solute carrier family 35, member F5 (Slc35f5), mRNA [NM_028787]	2249.7	2655.0	2685.3	1979.6	2769.8
19636	Slc44a5	Mus musculus solute carrier family 44, member 5, mRNA (cDNA clone MGC:99452 IMAGE:6417957), complete cds. [BC086641]	1.5	−1.0	−3.8	3.8	0.9
19727	Slc2a4	Mus musculus solute carrier family 2 (facilitated glucose transporter), member 4 (Slc2a4), mRNA [NM_009204]	3035.5	3872.4	4497.6	3142.1	4131.8
19759	Slc24a6	Mus musculus solute carrier family 24 (sodium/potassium/calcium exchanger), member 6 (Slc24a6), mRNA [NM_133221]	323.6	350.4	420.4	333.5	489.3
19764	Slc4a4	Mus musculus 4 days neonate male adipose cDNA, RIKEN full-length enriched library, clone:B430311M19 product:unclassifiable, full insert sequence. [AK163619]	1619.3	2130.3	1570.8	1942.1	2124.7
19788	Slc25a19	Mus musculus solute carrier family 25 (mitochondrial deoxynucleotide carrier), member 19 (Slc25a19), mRNA [NM_026071]	12023.1	9557.5	13391.8	11888.4	11227.9
19824	Slc37a3	Mus musculus solute carrier family 37 (glycerol-3-phosphate transporter), member 3 (Slc37a3), mRNA [NM_028123]	3199.9	3357.4	3614.6	3019.4	3884.9
19903	Slc6a17	Mus musculus solute carrier family 6 (neurotransmitter transporter), member 17 (Slc6a17), mRNA [NM_172271]	83.0	153.9	190.8	104.1	113.5
20185	Slc35e1	Mus musculus solute carrier family 35, member E1 (Slc35e1), mRNA [NM_177766]	81.7	50.1	70.2	104.4	65.1
20209	Slc32a1	Mus musculus solute carrier family 32 (GABA vesicular transporter), member 1 (Slc32a1), mRNA [NM_009508]	16.1	4.2	3.4	5.9	14.5
20229	Slc25a22	Mus musculus solute carrier family 25 (mitochondrial carrier, glutamate), member 22 (Slc25a22), mRNA [NM_026646]	426.4	445.8	650.4	402.2	347.7
20269	Slc7a14	Mus musculus solute carrier family 7 (cationic amino acid transporter, y+ system), member 14 (Slc7a14), mRNA [NM_172861]	14.2	18.1	10.0	6.2	6.0
20351	Slc17a5	Mus musculus solute carrier family 17 (anion/sugar transporter), member 5 (Slc17a5), mRNA [NM_172773]	3593.7	3589.9	4213.8	4047.8	4510.5
20358	Slc4a8	Mus musculus solute carrier family 4 (anion exchanger), member 8 (Slc4a8), mRNA [NM_021530]	120.9	118.8	173.0	107.8	114.4
20360	Slc30a5	Mus musculus solute carrier family 30 (zinc transporter), member 5 (Slc30a5), mRNA [NM_022885]	6460.6	6099.5	5861.2	5461.7	6680.7
20682	Slc7a13	Mus musculus solute carrier family 7, (cationic amino acid transporter, y+ system) member 13 (Slc7a13), mRNA [NM_028746]	39722.7	38659.2	41223.3	32970.7	34132.4
20687	Slc23a1	Mus musculus solute carrier family 23 (nucleobase transporters), member 1 (Slc23a1), mRNA [NM_011397]	3113.8	4724.5	4874.3	3625.4	4548.3
20721	Slc4a9	Mus musculus solute carrier family 4, sodium bicarbonate cotransporter, member 9 (Slc4a9), mRNA [NM_172830]	864.3	693.2	912.8	742.2	726.1
20757	Slc23a1	Mus musculus solute carrier family 23 (nucleobase transporters), member 1 (Slc23a1), mRNA [NM_011397]	4462.1	5820.9	7619.2	4794.6	6117.1
20770	Slc34a1	Mus musculus 0 day neonate kidney cDNA, RIKEN full-length enriched library, clone:D630036H03 product:unclassifiable, full insert sequence [AK085515]	4965.7	3768.4	4409.9	6442.2	4033.5
20942	Slc25a1	Mus musculus solute carrier family 25 (mitochondrial carrier, citrate transporter), member 1 (Slc25a1), mRNA [NM_153150]	4749.3	4494.9	6106.1	3854.4	4764.4
21054	Slc19a2	Mus musculus solute carrier family 19 (thiamine transporter), member 2 (Slc19a2), mRNA [NM_054087]	2262.8	2347.2	2927.6	1759.2	2274.6
21126	Slc16a1	Mus musculus solute carrier family 16 (monocarboxylic acid transporters), member 1 (Slc16a1), mRNA [NM_009196]	2745.4	2362.3	5978.3	3393.7	3141.9
21138	Slc45a2	Mus musculus solute carrier family 45, member 2 (Slc45a2), mRNA [NM_053077]	40.2	41.7	47.8	80.5	43.6
21143	Slc4a8	Mus musculus solute carrier family 4 (anion exchanger), member 8 (Slc4a8), mRNA [NM_021530]	179.3	264.2	374.5	223.2	228.4

21259	Slc25a33	Mus musculus solute carrier family 25, member 33 (Slc25a33), mRNA [NM_027460]	5214.1	5509.1	3826.9	3567.8	4915.2
21275	Abcc1	Mus musculus ATP-binding cassette, sub-family C (CFTR/MRP), member 1 (Abcc1), mRNA [NM_008576]	225.4	303.1	414.4	326.5	390.2
21290	Slc25a30	Mus musculus osteoclast-like cell cDNA, RIKEN full-length enriched library, clone:I420029E17 product:similar to Brain mitochondrial carrier protein-1 (BMCP-1) (Mitochondrial uncoupling protein 5) (UCP 5) (Solute carrier family 25, member 14) (UNQ791...	3030.3	2311.1	3328.8	3991.3	2966.6
21536	Slc26a8	Mus musculus solute carrier family 26, member 8 (Slc26a8), mRNA [NM_146076]	3.6	1.6	6.7	3.7	5.2
21553	Abca9	Mus musculus ATP-binding cassette transporter sub-family A member 9 (Abca9), mRNA [NM_147220]	75.7	88.0	95.2	75.5	99.0
21676	Abca8a	Mus musculus ATP-binding cassette, sub-family A (ABC1), member 8a (Abca8a), mRNA [NM_153145]	608.2	393.5	345.4	355.3	330.1
21842	Abca9	Mus musculus ATP-binding cassette transporter sub-family A member 9 (Abca9), mRNA [NM_147220]	4.9	9.8	6.7	10.2	15.8
21856	Slc7a4	Mus musculus solute carrier family 7 (cationic amino acid transporter, y+ system), member 4 (Slc7a4), mRNA [NM_144852]	188.7	183.9	272.7	223.8	288.6
21892	Abcg3	Mus musculus ATP-binding cassette, sub-family G (WHITE), member 3 (Abcg3), mRNA [NM_030239]	4.1	9.4	16.3	6.7	7.6
22019	Slc25a40	Mus musculus solute carrier family 25, member 40 (Slc25a40), mRNA [NM_178766]	45.4	84.8	52.6	70.4	83.6
22084	Slc29a4	Mus musculus solute carrier family 29 (nucleoside transporters), member 4 (Slc29a4), mRNA [NM_146257]	52.8	11.5	18.2	14.7	13.1
22134	Slc16a11	Mus musculus solute carrier family 16 (monocarboxylic acid transporters), member 11 (Slc16a11), mRNA [NM_153081]	10046.6	12836.7	12848.4	10270.2	16105.5
22184	Slc25a35	Mus musculus solute carrier family 25, member 35 (Slc25a35), mRNA [NM_028048]	333.0	438.2	548.6	390.7	551.6
22245	Slc25a36	Mus musculus solute carrier family 25, member 36 (Slc25a36), mRNA [NM_138756]	881.3	768.3	882.3	671.1	689.3
22525	Slc8a1	Mus musculus solute carrier family 8 (sodium/calcium exchanger), member 1 (Slc8a1), mRNA [NM_011406]	250.9	206.0	286.7	241.0	315.3
22631	Slc17a7	Mus musculus solute carrier family 17 (sodium-dependent inorganic phosphate cotransporter), member 7 (Slc17a7), mRNA [NM_182993]	0.5	3.3	−0.3	−1.2	−0.3
22665	Abce1	Mus musculus ATP-binding cassette, sub-family E (OABP), member 1 (Abce1), mRNA [NM_015751]	88.9	108.5	133.8	104.8	157.2
22756	Slc5a4b	Mus musculus solute carrier family 5 (neutral amino acid transporters, system A), member 4b (Slc5a4b), mRNA [NM_023219]	31.9	64.9	32.1	36.9	47.1
22868	Slc4a2	Mus musculus solute carrier family 4 (anion exchanger), member 2 (Slc4a2), mRNA [NM_009207]	2114.0	2127.1	2968.1	1830.8	2093.5
22912	Slc9a10	Mus musculus solute carrier family 9, isoform 10 (Slc9a10), mRNA [NM_198106]	6.4	1.7	4.8	6.9	16.0
22923	Slc6a15	Mus musculus adult male corpora quadrigemina cDNA, RIKEN full-length enriched library, clone:B230311C12 product:ORPHAN SODIUM- AND CHLORIDE-DEPENDENT NEUROTRANSMITTER TRANSPORTER NTT73 (ORPHAN TRANSPORTER V7-3) homolog [Homo sapiens], full insert...	−0.4	−2.1	−1.7	−2.0	52.3
22971	Slc11a2	Mus musculus solute carrier family 11 (proton-coupled divalent metal ion transporters), member 2 (Slc11a2), mRNA [NM_008732]	166.8	194.6	230.8	170.2	217.2
22992	Slc6a15	Mus musculus solute carrier family 6 (neurotransmitter transporter), member 15 (Slc6a15), mRNA [NM_175328]	1300.3	1574.2	1415.9	2499.3	3028.5
23087	Abca9	Mus musculus 0 day neonate skin cDNA, RIKEN full-length enriched library, clone:4631409B15 product:hypothetical protein, full insert sequence. [AK028451]	−2.4	0.6	−3.2	−0.7	−2.2
23126	Slc6a17	Mus musculus solute carrier family 6 (neurotransmitter transporter), member 17 (Slc6a17), mRNA [NM_172271]	75.5	54.0	72.2	40.4	59.7
23498	Slc6a13	Mus musculus solute carrier family 6 (neurotransmitter transporter, GABA), member 13 (Slc6a13), mRNA [NM_144512]	12160.6	12235.9	14490.6	14307.5	16125.1
23499	Slc25a43	PREDICTED: Mus musculus gene model 8, (NCBI), transcript variant 1 (Gm8), mRNA [XM_110743]	26.3	22.8	28.5	24.4	45.1
23583	Slc8a3	Mus musculus solute carrier family 8 (sodium/calcium exchanger), member 3, mRNA (cDNA clone MGC:63358 IMAGE:6837128), complete cds. [BC052435]	12.7	8.2	2.2	13.2	16.8
23722	Slc35d1	Mus musculus solute carrier family 35 (UDP-glucuronic acid/UDP-N-acetylgalactosamine dual transporter), member D1 (Slc35d1), mRNA [NM_177732]	1217.3	1444.2	1371.2	1505.4	1765.7
23791	Slc44a1	Mus musculus solute carrier family 44, member 1 (Slc44a1), mRNA [NM_133891]	400.9	420.2	510.3	382.8	506.5
23894	Slco2a1	Mus musculus solute carrier organic anion transporter family, member 2a1 (Slco2a1), mRNA [NM_033314]	786.8	1244.9	1049.5	936.5	1174.6
24016	Slc17a6	Mus musculus solute carrier family 17 (sodium-dependent inorganic phosphate cotransporter), member 6 (Slc17a6), mRNA [NM_080853]	23.6	14.6	17.5	9.5	11.8
24102	Slc16a5	PREDICTED: Mus musculus solute carrier family 16 (monocarboxylic acid transporters), member 5, transcript variant 1 (Slc16a5), mRNA [XM_126601]	137.4	103.3	107.1	171.5	142.2
24107	Abcc4	Mus musculus ATP-binding cassette, sub-family C (CFTR/MRP), member 4 (Abcc4), mRNA [NM_001033336]	2599.6	3425.4	3175.1	1771.2	2707.5
24176	Slc25a38	Mus musculus solute carrier family 25, member 38 (Slc25a38), mRNA [NM_144793]	4046.0	2671.6	3814.9	2688.3	3053.5
24210	Slc22a20	Mus musculus 15 days embryo head cDNA, RIKEN full-length enriched library, clone:D930013K24 product:unclassifiable, full insert sequence [AK053009]	0.9	1.7	−1.9	0.0	0.5
24273	Abcb7	PREDICTED: Mus musculus ATP-binding cassette, sub-family B (MDR/TAP), member 7, transcript variant 5 (Abcb7), mRNA [XM_907304]	1629.4	1423.8	1287.2	1547.0	1539.3
24285	Slc4a7	Mus musculus solute carrier family 4, sodium bicarbonate cotransporter, member 7 (Slc4a7), mRNA [NM_001033270]	3630.2	3690.3	2941.0	2575.6	3270.1
24296	Slc36a4	Mus musculus solute carrier family 36 (proton/amino acid symporter), member 4 (Slc36a4), mRNA [NM_172289]	13.7	16.7	19.6	16.4	25.8
24322	Slc14a2	Mus musculus solute carrier family 14 (urea transporter), member 2 (Slc14a2), transcript variant 2, mRNA [NM_030683]	856.6	620.5	776.8	431.8	576.2
24403	Slc12a9	Mus musculus solute carrier family 12 (potassium/chloride transporters), member 9 (Slc12a9), mRNA [NM_031406]	382.5	438.4	586.9	337.4	424.8
24414	Abcc9	Mus musculus ATP-binding cassette, sub-family C (CFTR/MRP), member 9 (Abcc9), transcript variant 2, mRNA [NM_021041]	213.2	342.0	235.1	161.4	215.8
24437	Slc6a7	Mus musculus solute carrier family 6 (neurotransmitter transporter, L-proline), member 7 (Slc6a7), mRNA [NM_201353]	37.2	23.3	31.8	23.0	17.6
24485	Slc17a6	Mus musculus solute carrier family 17 (sodium-dependent inorganic phosphate cotransporter), member 6 (Slc17a6), mRNA [NM_080853]	2.3	3.3	−0.3	4.5	4.3
24587	Slc18a3	Mus musculus solute carrier family 18 (vesicular monoamine), member 3 (Slc18a3), mRNA [NM_021712]	19.0	30.0	7.7	5.7	5.3
24668	Slc34a2	Mus musculus solute carrier family 34 (sodium phosphate), member 2 (Slc34a2), mRNA [NM_011402]	92.1	140.9	94.8	131.0	270.3
24694	Slc7a3	Mus musculus solute carrier family 7 (cationic amino acid transporter, y+ system), member 3 (Slc7a3), mRNA [NM_007515]	147.7	27.9	26.5	20.2	39.3
24859	Slc20a1	Mus musculus solute carrier family 20, member 1 (Slc20a1), mRNA [NM_015747]	289.1	260.2	311.4	222.5	269.1
24969	Slc38a2	Mus musculus 13 days embryo heart cDNA, RIKEN full-length enriched library, clone:D330050A21 product:solute carrier family 38, member 2, full insert sequence [AK164568]	47.4	47.9	61.3	49.7	51.2
24984	Abca3	Mus musculus ATP-binding cassette, sub-family A (ABC1), member 3 (Abca3), transcript variant 1, mRNA [NM_013855]	22337.0	17427.5	18052.3	20633.5	17764.9
25086	Slc27a4	Mus musculus solute carrier family 27 (fatty acid transporter), member 4 (Slc27a4), mRNA [NM_011989]	919.9	821.2	1096.7	875.0	908.6
25133	Slc1a5	Mus musculus solute carrier family 1 (neutral amino acid transporter), member 5 (Slc1a5), mRNA [NM_009201]	1939.6	2031.8	2253.3	1571.0	1583.5
25167	Slc16a7	Mus musculus 0 day neonate kidney cDNA, RIKEN full-length enriched library, clone:D630022A07 product:MONOCARBOXYLATE TRANSPORTER 2 (MCT 2) homolog [Mus musculus], full insert sequence [AK085398]	39.5	56.5	142.6	50.0	71.0
25256	Abca6	Mus musculus adult male aorta and vein cDNA, RIKEN full-length enriched library, clone:A530006M05 product:hypothetical protein, full insert sequence. [AK040652]	0.9	6.3	4.7	6.5	12.1
25257	Slc39a13	Mus musculus solute carrier family 39 (metal ion transporter), member 13 (Slc39a13), mRNA [NM_026721]	2211.4	2373.7	3355.1	1984.4	2203.8
25300	Slc23a2	Mus musculus solute carrier family 23 (nucleobase transporters), member 2 (Slc23a2), mRNA [NM_018824]	166.0	213.4	167.7	145.5	197.1
25391	Slc39a4	Mus musculus solute carrier family 39 (zinc transporter), member 4 (Slc39a4), mRNA [NM_028064]	37.6	37.3	50.1	22.7	34.0
25501	Slc22a2	Mus musculus solute carrier family 22 (organic cation transporter), member 2 (Slc22a2), mRNA [NM_013667]	31880.4	36964.2	38102.6	30726.0	38446.6
25698	Slc25a15	Mus musculus solute carrier family 25 (mitochondrial carrier ornithine transporter), member 15 (Slc25a15), mRNA [NM_011017]	1001.2	968.1	1016.6	1123.5	890.2
25705	Slc9a1	Mus musculus solute carrier family 9 (sodium/hydrogen exchanger), member 1 (Slc9a1), mRNA [NM_016981]	664.7	741.1	1032.2	593.9	719.0
25782	Slc22a18	Mus musculus solute carrier family 22 (organic cation transporter), member 18 (Slc22a18), transcript variant 2, mRNA [NM_001042760]	88800.7	97189.4	118096.3	81916.3	88522.5
25946	Slc45a1	Mus musculus 0 day neonate cerebellum cDNA, RIKEN full-length enriched library, clone:C230078B22 product:Proton-associated sugar transporter A (PAST-A) (Deleted in neuroblastoma 5 protein homolog) (DNb-5 homolog), full insert sequence [AK082651]	66.1	62.6	89.3	67.5	110.3
25974	Slc36a3	Mus musculus solute carrier family 36 (proton/amino acid symporter), member 3 (Slc36a3), mRNA [NM_172258]	31.9	27.8	24.4	21.2	23.4
25984	Abcc12	Mus musculus ATP-binding cassette, sub-family C (CFTR/MRP), member 12 (Abcc12), mRNA [NM_172912]	0.3	5.2	−3.2	1.5	−0.7
26002	Abcb4	Mus musculus ATP-binding cassette, sub-family B (MDR/TAP), member 4 (Abcb4), mRNA [NM_008830]	599.8	716.5	665.8	3271.5	3717.4
26026	Slc14a1	Mus musculus solute carrier family 14 (urea transporter), member 1 (Slc14a1), mRNA [NM_028122]	0.5	24.1	66.3	24.3	44.0
26107	Slc7a9	Mus musculus solute carrier family 7 (cationic amino acid transporter, y+ system), member 9 (Slc7a9), mRNA [NM_021291]	28609.7	30436.6	35292.2	23296.0	28385.7
26168	Abcb5	PREDICTED: Mus musculus ATP-binding cassette, sub-family B (MDR [XM_001002680]	0.4	0.7	0.1	−0.1	−1.0
26227	Slc1a1	Mus musculus solute carrier family 1 (neuronal/epithelial high affinity glutamate transporter, system Xag), member 1 (Slc1a1), mRNA [NM_009199]	13423.7	12616.0	15416.8	13623.6	15785.4
26498	Abcg4	Mus musculus ATP-binding cassette, sub-family G (WHITE), member 4 (Abcg4), mRNA [NM_138955]	10.9	16.5	18.0	9.1	20.7
26549	Slc20a2	Mus musculus solute carrier family 20, member 2 (Slc20a2), mRNA [NM_011394]	794.1	806.5	801.6	945.1	924.4
26799	Slc2a4	Mus musculus solute carrier family 2 (facilitated glucose transporter), member 4 (Slc2a4), mRNA [NM_009204]	3155.7	3987.2	4450.5	3198.1	3761.8
26835	Slc41a1	Mus musculus solute carrier family 41, member 1 (Slc41a1), mRNA [NM_173865]	578.5	637.8	649.9	420.5	510.9
27026	Slc3a2	Mus musculus solute carrier family 3 (activators of dibasic and neutral amino acid transport), member 2 (Slc3a2), mRNA [NM_008577]	35565.6	25679.8	37203.1	25269.5	24229.9
27092	Slc13a5	Mus musculus solute carrier family 13 (sodium-dependent citrate transporter), member 5 (Slc13a5), mRNA [NM_001004148]	15.5	14.3	20.8	16.4	14.8
27177	Slc30a9	Mus musculus solute carrier family 30 (zinc transporter), member 9 (Slc30a9), mRNA [NM_178651]	8195.9	9635.1	9159.3	9482.1	9918.4
27237	Slc26a7	Mus musculus 2 days pregnant adult female ovary cDNA, RIKEN full-length enriched library, clone:E330015I04 product:PUTATIVE ANION TRANSPORTER homolog [Homo sapiens], full insert sequence [AK054327]	57.6	111.7	109.6	100.4	126.0
27403	Slc19a1	Mus musculus solute carrier family 19 (sodium/hydrogen exchanger), member 1 (Slc19a1), mRNA [NM_031196]	22155.9	25482.7	28937.9	19694.2	22384.2
27433	Abcg1	Mus musculus ATP-binding cassette, sub-family G (WHITE), member 1 (Abcg1), mRNA [NM_009593]	575.9	536.6	544.3	413.8	430.0
27487	Slc25a5	Mus musculus solute carrier family 25 (mitochondrial carrier, adenine nucleotide translocator), member 5 (Slc25a5), mRNA [NM_007451]	208530.7	215243.7	228760.1	161182.9	198401.3
27642	Slc38a2	Mus musculus solute carrier family 38, member 2 (Slc38a2), mRNA [NM_175121]	300.0	276.5	358.4	330.7	357.9
27790	Abca17	Mus musculus ATP-binding cassette, sub-family A (ABC1), member 17 (Abca17), mRNA [NM_001031621]	23.6	17.0	19.6	16.3	19.3
27806	Abcc8	Mus musculus ATP-binding cassette, sub-family C (CFTR/MRP), member 8 (Abcc8), mRNA [NM_011510]	8.9	2.1	14.9	0.2	−1.1
27977	Abca2	Mus musculus ATP-binding cassette, sub-family A (ABC1), member 2 (Abca2), mRNA [NM_007379]	675.2	805.4	1065.5	531.9	658.6
28015	Slc8a1	Mus musculus solute carrier family 8 (sodium/calcium exchanger), member 1 (Slc8a1), mRNA [NM_011406]	326.9	400.8	378.7	412.0	554.8
28096	Slc7a8	Mus musculus solute carrier family 7 (cationic amino acid transporter, y+ system), member 8 (Slc7a8), mRNA [NM_016972]	9242.5	7780.7	9514.5	8182.0	7952.4
28169	Slc33a1	Mus musculus solute carrier family 33 (acetyl-CoA transporter), member 1 (Slc33a1), mRNA [NM_015728]	2149.5	2476.4	1742.2	2688.5	3289.4
28238	Slc9a6	Mus musculus solute carrier family 9 (sodium/hydrogen exchanger), isoform 6 (Slc9a6), mRNA [NM_172780]	1092.7	1195.9	1489.8	981.8	1210.7
28285	Slc27a3	Mus musculus solute carrier family 27 (fatty acid transporter), member 3 (Slc27a3), mRNA [NM_011988]	109.2	119.1	212.2	111.4	132.5
28383	Slc5a4b	Mus musculus solute carrier family 5 (neutral amino acid transporters, system A), member 4b (Slc5a4b), mRNA [NM_023219]	4.8	10.3	11.1	11.7	17.0
28479	Slc39a3	Mus musculus solute carrier family 39 (zinc transporter), member 3 (Slc39a3), mRNA [NM_134135]	1086.3	1098.0	1649.8	887.0	1064.2
28568	Slc11a1	Mus musculus solute carrier family 11 (proton-coupled divalent metal ion transporters), member 1 (Slc11a1), mRNA [NM_013612]	190.3	165.5	226.0	102.1	151.4
28746	Slc25a4	Mus musculus solute carrier family 25 (mitochondrial carrier, adenine nucleotide translocator), member 4 (Slc25a4), mRNA [NM_007450]	73568.2	83900.9	88310.2	58535.5	77927.3
28846	Abcd3	Mus musculus ATP-binding cassette, sub-family D (ALD), member 3 (Abcd3), mRNA [NM_008991]	782.2	1226.0	691.8	1084.5	1154.5
28994	Abcd4	Mus musculus ATP-binding cassette, sub-family D (ALD), member 4 (Abcd4), mRNA [NM_008992]	4575.3	6297.2	6205.6	4207.3	5220.8
29016	Slc25a29	Mus musculus solute carrier family 25 (mitochondrial carrier, palmitoylcarnitine transporter), member 29 (Slc25a29), mRNA [NM_181328]	246.9	224.4	341.7	209.6	231.7
29034	Slc2a3	M.musculus mRNA for glucose transporter type 3. [X69698]	0.6	0.9	0.6	−2.1	0.6
29055	Abcc6	Mus musculus ATP-binding cassette, sub-family C (CFTR/MRP), member 6 (Abcc6), mRNA [NM_018795]	113.5	102.2	127.1	85.2	100.4
29065	Slc44a2	Mus musculus solute carrier family 44, member 2 (Slc44a2), mRNA [NM_152808]	789.0	728.7	735.7	564.4	655.3
29097	Abcb7	Mus musculus bone marrow macrophage cDNA, RIKEN full-length enriched library, clone:I830078I15 product:ATP-binding cassette, sub-family B (MDR/TAP), member 7, full insert sequence. [AK152535]	445.3	393.6	487.0	354.7	391.6

29127	Slc6a16	PREDICTED: Mus musculus solute carrier family 6, member 16 (LOC381884), mRNA [XM_355900]	128.7	89.4	117.7	62.6	85.1
29158	Slc25a29	Mus musculus solute carrier family 25 (mitochondrial carrier, palmitoylcarnitine transporter), member 29 (Slc25a29), mRNA [NM_181328]	486.3	586.3	788.0	454.7	537.9
29208	Abcb1b	Mus musculus ATP-binding cassette, sub-family B (MDR/TAP), member 1B (Abcb1b), mRNA [NM_011075]	22.7	26.3	12.5	171.7	161.1
29269	Slc35c1	Mus musculus solute carrier family 35, member C1 (Slc35c1), transcript variant 1, mRNA [NM_211358]	5348.7	5648.4	6169.9	4363.9	5132.4
29275	Slc35f4	Mus musculus 16 days embryo head cDNA, RIKEN full-length enriched library, clone:C130023D01 product:hypothetical protein, full insert sequence. [AK047949]	3.2	0.5	2.6	−1.3	2.6
29391	Slc19a3	Mus musculus solute carrier family 19 (sodium/hydrogen exchanger), member 3 (Slc19a3), mRNA [NM_030556]	262.6	264.9	295.0	238.9	255.7
29450	Slc9a8	Mus musculus solute carrier family 9 (sodium/hydrogen exchanger), member 8 (Slc9a8), transcript variant 1, mRNA [NM_148929]	396.5	408.1	515.4	419.9	483.7
29469	Slc35f2	Mus musculus solute carrier family 35, member F2 (Slc35f2), mRNA [NM_028060]	765.8	858.9	1271.6	693.9	831.1
29560	Slc35d3	Mus musculus solute carrier family 35, member D3 (Slc35d3), mRNA [NM_029529]	8.9	4.5	6.0	3.0	7.3
29645	Slc25a17	Mus musculus solute carrier family 25 (mitochondrial carrier, peroxisomal membrane protein), member 17 (Slc25a17), mRNA [NM_011399]	4394.3	4357.7	4741.3	4882.1	4958.9
29705	Abcb10	Mus musculus ATP-binding cassette, sub-family B (MDR/TAP), member 10 (Abcb10), nuclear gene encoding mitochondrial protein, mRNA [NM_019552]	816.0	775.9	850.9	639.3	665.9
29717	Slc25a36	Mus musculus 16 days neonate thymus cDNA, RIKEN full-length enriched library, clone:A130013B19 product:hypothetical protein, full insert sequence. [AK037383]	920.2	1021.0	1085.2	656.2	860.8
29784	Slc10a1	Mus musculus solute carrier family 10 (sodium/bile acid cotransporter family), member 1 (Slc10a1), mRNA [NM_011387]	34.8	35.7	43.3	33.2	40.5
29793	Slc4a1ap	Mus musculus solute carrier family 4 (anion exchanger), member 1, adaptor protein (Slc4a1ap), mRNA [NM_009206]	209.2	230.8	257.2	258.1	205.0
29840	Slc41a1	Mus musculus solute carrier family 41, member 1 (Slc41a1), mRNA [NM_173865]	65.5	69.7	88.2	57.7	73.8
29927	Slc19a3	Mus musculus cDNA fis, clone TRACH2007674,weakly similar to FOLATE-LIKE TRANSPORTER [AK098112]	573.7	726.3	826.8	562.2	651.1
30081	Slc39a14	Mus musculus solute carrier family 39 (zinc transporter), member 14 (Slc39a14), mRNA [NM_144808]	296.2	190.9	299.8	283.2	307.9
30091	Slc25a12	Mus musculus solute carrier family 25 (mitochondrial carrier, Aralar), member 12 (Slc25a12), mRNA [NM_172436]	6907.8	7795.5	9338.1	5945.0	7150.1
30262	Slc6a18	Mus musculus solute carrier family 6 (neurotransmitter transporter), member 18 (Slc6a18), mRNA [NM_001040692]	8553.1	8664.8	11908.1	10329.3	8009.0
30291	Slc1a3	Mus musculus adult male corpora quadrigemina cDNA, RIKEN full-length enriched library, clone:B230307E07 product:similar to EXCITATORY AMINO ACID TRANSPORTER 1 (SODIUM-DEPENDENT GLUTAMATE/ASPARTATE TRANSPORTER) (HIGH-AFFINITY NEURONAL GLUTAMATE…	11.9	3.7	8.2	27.3	5.1
30493	Slc22a12	Mus musculus solute carrier family 22 (organic anion/cation transporter), member 12 (Slc22a12), mRNA [NM_009203]	13353.7	9803.5	11642.0	11430.0	10311.6
30924	Slc30a1	Mus musculus solute carrier family 30 (zinc transporter), member 1 (Slc30a1), mRNA [NM_009579]	4088.9	3478.2	3624.0	3163.1	3395.0
30973	Slc22a14	Mus musculus solute carrier family 22 (organic cation transporter), member 14 (Slc22a14), mRNA [NM_001037749]	−0.7	1.3	1.6	2.1	0.5
30975	Slc22a1	Mus musculus solute carrier family 22 (organic cation transporter), member 1 (Slc22a1), mRNA [NM_009202]	33547.2	31176.2	45082.8	28272.1	30447.2
31006	Slc6a18	Mus musculus solute carrier family 6 (neurotransmitter transporter), member 18 (Slc6a18), mRNA [NM_001040692]	12072.3	8721.8	14106.5	12529.8	10130.4
31152	Slc35f4	Mus musculus solute carrier family 35, member F4 (Slc35f4), mRNA [NM_029238]	−0.5	6.8	4.4	5.9	6.8
31220	Slc18a1	Mus musculus 7 days embryo whole body cDNA, RIKEN full-length enriched library, clone:C430018J19 product:CHROMAFFIN GRANULE AMINE TRANSPORTER (VESICULAR AMINE TRANSPORTER 1) (VAT1) homolog [Rattus norvegicus], full insert sequence. [AK049514]	23.3	41.6	43.0	23.6	44.7
31226	Slc7a2	Mus musculus solute carrier family 7 (cationic amino acid transporter, y+ system), member 2 (Slc7a2), transcript variant 1, mRNA [NM_007514]	10.2	4.9	5.0	7.0	7.1
31272	Slco1a4	Mus musculus solute carrier organic anion transporter family, member 1a4 (Slco1a4), mRNA [NM_030687]	30.3	28.2	47.8	30.1	48.6
31511	Slc25a37	Mus musculus 12 days embryo embryonic body between diaphragm region and neck cDNA, RIKEN full-length enriched library, clone:9430065F12 product:MITOCHONDRIAL CARRIER-LIKE PROTEIN homolog [Mus musculus], full insert sequence. [AK034948]	2.9	−2.5	−1.8	−3.8	−2.8
31540	Slc25a3	Mus musculus adult male brain cDNA, RIKEN full-length enriched library, clone:3526402M16 product:solute carrier family 25 (mitochondrial carrier; adenine nucleotide translocator), member 3, full insert sequence. [AK028313]	127.6	169.9	199.4	128.6	181.0
31631	Slco1c1	Mus musculus solute carrier organic anion transporter family, member 1c1 (Slco1c1), mRNA [NM_021471]	5.8	8.1	5.4	3.6	4.7
31636	Slc7a11	Mus musculus solute carrier family 7 (cationic amino acid transporter, y+ system), member 11 (Slc7a11), mRNA [NM_011990]	−0.4	−1.8	−3.0	−0.5	−1.1
31655	Slc25a28	Mus musculus solute carrier family 25, member 28 (Slc25a28), mRNA [NM_145156]	3709.5	4119.5	6042.2	3546.7	3902.3
31795	Slc25a40	Mus musculus adult male diencephalon cDNA, RIKEN full-length enriched library, clone:9330005I01 product:similar to Mitochondrial carrier family protein [Homo sapiens], full insert sequence. [AK136770]	386.1	485.5	477.6	325.4	418.6
31938	Slc26a3	Mus musculus solute carrier family 26, member 3, mRNA (cDNA clone IMAGE:4989186), complete cds. [BC037066]	10.6	11.9	10.6	10.0	9.5
32037	Slc26a4	Mus musculus solute carrier family 26, member 4 (Slc26a4), mRNA [NM_011867]	17327.1	16476.3	17602.3	11646.9	16390.2
32047	Slc3a2	Mus musculus solute carrier family 3 (activators of dibasic and neutral amino acid transport), member 2 (Slc3a2), mRNA [NM_008577]	532.4	597.1	943.0	401.0	483.7
32177	Slc25a17	Mus musculus solute carrier family 25 (mitochondrial carrier, peroxisomal membrane protein), member 17 (Slc25a17), mRNA [NM_011399]	3453.3	4426.9	4142.1	2023.4	3721.4
32210	Slc25a11	Mus musculus solute carrier family 25 (mitochondrial carrier oxoglutarate carrier), member 11 (Slc25a11), mRNA [NM_024211]	46778.5	50214.7	56477.4	35421.3	54238.6
32293	Slc35f3	Mus musculus solute carrier family 35, member F3 (Slc35f3), mRNA [NM_175434]	1497.3	2617.7	3089.8	1242.5	3064.2
32330	Abcb6	Mus musculus ATP-binding cassette, sub-family B (MDR/TAP), member 6 (Abcb6), mRNA [NM_023732]	2960.4	2837.2	4096.9	2678.6	2805.6
32374	Slc2a10	Mus musculus solute carrier family 2 (facilitated glucose transporter), member 10 (Slc2a10), mRNA [NM_130451]	22.2	18.1	25.6	19.4	18.9
32425	Slc9a8	Mus musculus 16 days neonate thymus cDNA, RIKEN full-length enriched library, clone:A130012L18 product:solute carrier family 9 (sodium/hydrogen exchanger), member 8, full insert sequence. [AK037377]	24.0	23.9	28.9	17.3	15.7
32473	Abca5	Mus musculus 16 days neonate cerebellum cDNA, RIKEN full-length enriched library, clone:9630020L03 product:weakly similar to ATP-BINDING CASSETTE PROTEIN [Homo sapiens], full insert sequence. [AK035955]	−0.6	−0.7	0.3	2.5	0.2
32533	Abcg8	Mus musculus ATP-binding cassette, sub-family G (WHITE), member 8 (Abcg8), mRNA [NM_026180]	−1.1	−0.1	2.6	−1.7	−1.3
32697	Slc25a3	Mus musculus solute carrier family 25 (mitochondrial carrier, phosphate carrier), member 3 (Slc25a3), mRNA [NM_133668]	47892.2	43411.4	54236.8	31126.8	42284.7
32718	Slc26a6	Mus musculus solute carrier family 26, member 6 (Slc26a6), mRNA [NM_134420]	481.1	469.3	477.2	404.8	515.5
32754	Slc16a4	Mus musculus solute carrier family 16 (monocarboxylic acid transporters), member 4 (Slc16a4), mRNA [NM_146136]	2101.4	2338.8	2249.9	1822.4	2511.5
32756	Slco4a1	Mus musculus solute carrier organic anion transporter family, member 4a1 (Slco4a1), mRNA [NM_148933]	451.5	441.3	703.5	471.6	487.1
32891	Slc10a6	Mus musculus solute carrier family 10 (sodium/bile acid cotransporter family), member 6 (Slc10a6), mRNA [NM_029415]	331.2	168.3	95.0	76.1	76.5
32912	Slc35d2	Mus musculus solute carrier family 35, member D2 (Slc35d2), mRNA [NM_001001321]	2660.7	2946.6	3627.7	2401.4	3627.3
32926	Abcd3	Mus musculus ATP-binding cassette, sub-family D (ALD), member 3 (Abcd3), mRNA [NM_008991]	1001.7	1319.2	883.9	1303.6	1370.8
32933	Abca4	Mus musculus ATP-binding cassette, sub-family A (ABC1), member 4 (Abca4), mRNA [NM_007378]	60.7	92.5	104.7	90.9	104.7
32948	Slc6a13	Mus musculus solute carrier family 6 (neurotransmitter transporter, GABA), member 13 (Slc6a13), mRNA [NM_144512]	16206.7	16557.9	17726.9	17125.8	18100.4
33081	Slc7a14	Mus musculus solute carrier family 7 (cationic amino acid transporter, y+ system), member 14 (Slc7a14), mRNA [NM_172861]	5.5	4.1	3.4	2.2	4.2
33102	Slc35b2	Mus musculus solute carrier family 35, member B2 (Slc35b2), mRNA [NM_028662]	7358.5	9911.3	12746.9	7380.3	10770.7
33143	Slc36a1	Mus musculus adult male thymus cDNA, RIKEN full-length enriched library, clone:5830411H19 product:unclassifiable, full insert sequence [AK017918]	3600.5	3435.5	3959.7	2734.6	3259.0
33158	Slc38a5	Mus musculus solute carrier family 38, member 5 (Slc38a5), mRNA [NM_172479]	7.9	37.5	4.9	4.5	12.5
33161	Slc26a1	Mus musculus solute carrier family 26 (sulfate transporter), member 1 (Slc26a1), mRNA [NM_174870]	4008.4	4901.3	5950.4	3979.7	4443.6
33249	Slc2a5	Mus musculus solute carrier family 2 (facilitated glucose transporter), member 5 (Slc2a5), mRNA [NM_019741]	7542.5	13298.4	12316.2	8726.6	12275.2
33264	Slc16a10	Mus musculus solute carrier family 16 (monocarboxylic acid transporters), member 10 (Slc16a10), mRNA [NM_028247]	139.7	140.0	191.6	114.5	138.7
33365	Slc35d2	Mus musculus solute carrier family 35, member D2 (Slc35d2), mRNA [NM_001001321]	2644.5	3538.1	3930.3	2773.0	3879.5
33470	Slc23a3	Mus musculus solute carrier family 23 (nucleobase transporters), member 3 (Slc23a3), mRNA [NM_194333]	3211.6	3656.8	5264.4	3782.5	5287.8
33535	Slc2a4	Mus musculus solute carrier family 2 (facilitated glucose transporter), member 4 (Slc2a4), mRNA [NM_009204]	2641.4	3734.7	4039.4	2877.3	3643.2
33549	Abca3	Mus musculus ATP-binding cassette, sub-family A (ABC1), member 3 (Abca3), transcript variant 1, mRNA [NM_013855]	25819.4	20422.4	16128.3	19840.5	17045.7
33574	Slco2b1	Mus musculus solute carrier organic anion transporter family, member 2b1 (Slco2b1), mRNA [NM_175316]	1972.0	1929.6	1688.5	1424.5	1783.7
33599	Slco1a1	Mus musculus strain CAST [AY195869]	32607.7	35251.9	36047.4	27107.8	29099.4
33607	Abca14	Mus musculus ATP-binding cassette, sub-family A (ABC1), member 14 (Abca14), mRNA [NM_026458]	11.6	5.7	5.7	3.6	5.7
33630	Slc8a1	Mus musculus 16 days embryo head cDNA, RIKEN full-length enriched library, clone:C130038C08 product:solute carrier family 8 (sodium/calcium exchanger), member 1, full insert sequence. [AK048160]	0.7	4.6	−2.9	−1.6	−2.4
33650	Slc35a3	Mus musculus 16 days embryo head cDNA, RIKEN full-length enriched library, clone:C130098A19 product:weakly solute carrier family 35 (UDP-N-acetylglucosamine (UDP-GlcNAc) transporter), member 3, full insert sequence. [AK082040]	127.0	96.2	85.4	128.2	95.5
33743	Slc16a7	Mus musculus solute carrier family 16 (monocarboxylic acid transporters), member 7 (Slc16a7), mRNA [NM_011391]	2907.0	3365.3	4689.4	3102.2	3873.1
33750	Abca2	Mus musculus ATP-binding cassette, sub-family A (ABC1), member 2 (Abca2), mRNA [NM_007379]	733.9	952.0	1357.7	560.4	747.4
33871	Abcf2	Mus musculus ATP-binding cassette, sub-family F (GCN20), member 2 (Abcf2), mRNA [NM_013853]	1629.8	1553.7	2419.4	1577.3	1784.9
33951	Slc35a3	Mus musculus solute carrier family 35 (UDP-N-acetylglucosamine (UDP-GlcNAc) transporter), member 3 (Slc35a3), mRNA [NM_144902]	684.4	854.9	906.7	817.1	955.0
33952	Slc36a1	Mus musculus solute carrier family 36 (proton/amino acid symporter), member 1 (Slc36a1), mRNA [NM_153139]	29.4	26.1	44.6	24.9	33.7
33970	Slc41a2	Mus musculus solute carrier family 41, member 2 (Slc41a2), mRNA [NM_177388]	1176.5	1353.7	1625.0	1224.2	1549.6
34010	Slc2a3	Mus musculus cDNA, RIKEN full-length enriched library, clone:M5C1003G12 product:solute carrier family 2 (facilitated glucose transporter), member 3, full insert sequence. [AK147258]	0.4	−2.4	−3.8	−4.4	−2.0
34086	Abcb4	Mus musculus ATP-binding cassette, sub-family B (MDR/TAP), member 4 (Abcb4), mRNA [NM_008830]	2.7	6.3	50.7	33.6	28.7
34089	Abcb10	Mus musculus 10 days embryo whole body cDNA, RIKEN full-length enriched library, clone:2610027L15 product:ATP-binding cassette, sub-family B (MDR/TAP), member 10, full insert sequence. [AK011569]	9.5	6.4	9.6	5.1	7.5
34188	Slc16a9	Mus musculus solute carrier family 16 (monocarboxylic acid transporters), member 9 (Slc16a9), mRNA [NM_025807]	9731.9	14302.1	13251.0	8577.1	12190.6
34226	Slc9a4	Mus musculus solute carrier family 9 (sodium/hydrogen exchanger), member 4 (Slc9a4), mRNA [NM_177084]	24.7	23.1	6.9	8.8	9.4
34229	Slc15a2	Mus musculus solute carrier family 15 (H+/peptide transporter), member 2 (Slc15a2), mRNA [NM_021301]	11835.2	13079.0	15679.3	11757.7	14196.3
34326	Slc16a12	Mus musculus solute carrier family 16 (monocarboxylic acid transporters), member 12 (Slc16a12), mRNA [NM_172838]	6054.3	6480.1	5430.9	4631.3	6287.2
34375	Slc22a4	Mus musculus solute carrier family 22 (organic cation transporter), member 4 (Slc22a4), mRNA [NM_019687]	5839.4	7570.3	9120.5	6070.1	7183.0
34417	Slco1b2	Mus musculus solute carrier organic anion transporter family, member 1b2 (Slco1b2), transcript variant 2, mRNA [NM_178235]	41.4	29.3	46.4	88.3	93.0
34535	Slc24a5	Mus musculus solute carrier family 24, member 5 (Slc24a5), mRNA [NM_175034]	20.1	30.7	54.2	30.2	46.2
34617	Slc12a5	Mus musculus solute carrier family 12, member 5 (Slc12a5), mRNA [NM_020333]	413.2	423.9	531.5	346.0	403.0
34698	Abcc9	Mus musculus ATP-binding cassette, sub-family C (CFTR/MRP), member 9 (Abcc9), transcript variant 2, mRNA [NM_021041]	41.1	52.2	55.3	43.2	47.7
34781	Slc35b3	Mus musculus solute carrier family 35, member B3 (Slc35b3), mRNA [NM_134060]	2349.0	3010.1	3851.0	2363.4	3704.4
34801	Slc17a3	Mus musculus solute carrier family 17 (sodium phosphate), member 3 (Slc17a3), mRNA [NM_134069]	874.0	650.9	759.7	873.7	756.0
34803	Abcb7	Mus musculus ATP-binding cassette, sub-family B (MDR [BC080769]	−0.1	−0.1	1.2	2.8	0.3
34885	Slc40a1	Mus musculus solute carrier family 40 (iron-regulated transporter), member 1 (Slc40a1), mRNA [NM_016917]	5586.9	5313.3	6810.1	3943.1	5227.2
34887	Slc44a2	Mus musculus solute carrier family 44, member 2 (Slc44a2), mRNA [NM_152808]	728.8	784.9	990.1	633.1	703.3
34937	Slc1a2	Mus musculus solute carrier family 1 (glial high affinity glutamate transporter), member 2 (Slc1a2), transcript variant 1, mRNA [NM_001077514]	56.1	51.2	67.6	40.3	46.0
35070	Slc35b4	Mus musculus solute carrier family 35, member B4 (Slc35b4), mRNA [NM_021435]	3792.6	4086.1	4384.1	3769.1	3998.2
35372	Slc5a11	Mus musculus solute carrier family 5 (sodium/glucose cotransporter), member 11 (Slc5a11), mRNA [NM_146198]	16110.1	18963.0	21340.7	14558.9	16867.2
35394	Slc35e3	Mus musculus solute carrier family 35, member E3 (Slc35e3), mRNA [NM_029875]	13536.1	11767.7	13278.3	11983.5	12820.4
35431	Slc45a4	Mus musculus solute carrier family 45, member 4, mRNA (cDNA clone MGC:67529 IMAGE:5720940), complete cds. [BC056501]	4747.8	4148.1	5184.0	4469.2	4733.8
35469	Slc16a14	Mus musculus adult male hippocampus cDNA, RIKEN full-length enriched library, clone:C630003C14 product:similar to CDNA FLJ30794 FIS, CLONE FEBRA2001093, WEAKLY SIMILAR TO MONOCARBOXYLATE TRANSPORTER 4 [Homo sapiens], full insert sequence [AK049846]	223.9	185.4	147.9	164.5	159.0

35854	Slc6a9	Mus musculus solute carrier family 6 (neurotransmitter transporter, glycine), member 9 (Slc6a9), mRNA [NM_008135]	4307.7	4833.0	5396.8	4711.6	5906.0
35951	Slc16a4	Mus musculus solute carrier family 16 (monocarboxylic acid transporters), member 4 (Slc16a4), mRNA [NM_146136]	9364.1	7676.2	8572.1	6030.8	8342.0
35958	Abcg8	Mus musculus ATP-binding cassette, sub-family G (WHITE), member 8 (Abcg8), mRNA [NM_026180]	10.8	5.3	11.5	10.1	6.6
35990	Slc20a2	Mus musculus solute carrier family 20, member 2 (Slc20a2), mRNA [NM_011394]	515.5	562.4	569.3	716.6	712.0
36014	Slc25a24	Mus musculus solute carrier family 25 (mitochondrial carrier, phosphate carrier), member 24 (Slc25a24), mRNA [NM_172685]	236.6	209.6	207.1	162.5	232.1
36140	Slc39a1	M.musculus partial cochlear mRNA (clone 301A6). [Z78150]	26.4	32.0	44.8	20.8	34.6
36174	Slc7a5	Mus musculus solute carrier family 7 (cationic amino acid transporter, y+ system), member 5 (Slc7a5), mRNA [NM_011404]	99.1	102.6	77.1	176.2	99.6
36288	Slco4c1	AV024403 Mus musculus adult C57BL [AV024403]	347.7	544.4	781.6	726.1	718.4
36291	Abca5	ATP-binding cassette, sub-family A (ABC1), member 5 [Source:MarkerSymbol;Acc:MGI:2386607] [ENSMUST00000103052]	891.3	1029.2	1045.8	775.5	814.2
36361	Abcf1	Mus musculus ATP-binding cassette, sub-family F (GCN20), member 1 (Abcf1), mRNA [NM_013854]	1934.6	1946.7	1611.8	2319.8	2131.0
36454	Slco1a6	Mus musculus solute carrier organic anion transporter family, member 1a6 (Slco1a6), mRNA [NM_023718]	77048.2	59717.0	60378.2	58517.5	61568.9
36480	Slc30a10	Mus musculus solute carrier family 30, member 10 (Slc30a10), mRNA [NM_001033286]	0.7	0.0	−0.8	−0.7	−1.5
36560	Slc16a1	Mus musculus solute carrier family 16 (monocarboxylic acid transporters), member 1 (Slc16a1), mRNA [NM_009196]	47.5	53.6	111.6	100.7	115.0
36654	Slc35a4	Mus musculus solute carrier family 35, member A4 (Slc35a4), mRNA [NM_026404]	1019.0	948.7	1306.9	975.5	1075.5
36674	Slc35a5	Mus musculus solute carrier family 35, member A5 (Slc35a5), mRNA [NM_028756]	31.6	73.0	62.6	61.1	91.9
36853	Slc25a37	Mus musculus solute carrier family 25, member 37 (Slc25a37), mRNA [NM_026331]	3637.4	3456.9	3378.7	3569.3	4609.6
36862	Slc25a46	Mus musculus solute carrier family 25, member 46 (Slc25a46), mRNA [NM_026165]	5617.3	5445.9	6347.9	4394.7	5567.7
36909	Slc27a4	Mus musculus solute carrier family 27 (fatty acid transporter), member 4 (Slc27a4), mRNA [NM_011989]	63.2	49.4	76.5	63.1	63.9
36923	Slc25a31	Mus musculus solute carrier family 25 (mitochondrial carrier; adenine nucleotide translocator), member 31 (Slc25a31), mRNA [NM_178386]	1.9	0.8	5.7	4.4	3.0
36933	Slc22a6	solute carrier family 22 (organic anion transporter), member 6 [Source:MarkerSymbol;Acc:MGI:892001] [ENSMUST00000010250]	6976.8	6245.5	6430.3	6919.0	6447.1
37011	Slc35e1	Mus musculus 1 month neonate cerebellum cDNA, RIKEN full-length enriched library, clone:G630022L16 product:unclassifiable, full insert sequence. [AK090231]	91.5	163.2	90.0	80.2	65.3
37037	Slc6a3	Mus musculus solute carrier family 6 (neurotransmitter transporter, dopamine), member 3 (Slc6a3), mRNA [NM_010020]	15.1	13.3	10.6	16.9	16.0
37110	Slc6a20	Mus musculus solute carrier family 6 (neurotransmitter transporter), member 20 (Slc6a20), mRNA [NM_011731]	13089.3	11764.7	13141.7	12483.3	13461.4
37269	Slc12a3	Mus musculus solute carrier family 12, member 3 (Slc12a3), mRNA [NM_019415]	5666.7	4276.8	6476.9	5425.7	5148.1
37289	Slc5a1	Mus musculus solute carrier family 5 (sodium/glucose cotransporter), member 1 (Slc5a1), mRNA [NM_019810]	1426.0	1103.2	1213.9	1577.1	1497.2
37463	Slc9a6	Mus musculus solute carrier family 9 (sodium/hydrogen exchanger), isoform 6 (Slc9a6), mRNA [NM_172780]	886.1	1005.6	989.1	891.9	1112.7
37523	Slc25a16	Mus musculus solute carrier family 25 (mitochondrial carrier, Graves disease autoantigen), member 16 (Slc25a16), mRNA [NM_175194]	2762.8	3449.6	3036.2	2055.1	2795.7
37578	Slc13a1	Mus musculus 10 days neonate cerebellum cDNA, RIKEN full-length enriched library, clone:B930093I16 product:solute carrier family 13 (sodium [AK047580]	18.5	8.6	13.1	13.3	147.3
37582	Slc5a9	solute carrier family 5 (sodium/glucose cotransporter), member 9 [Source:MarkerSymbol;Acc:MGI:2140201] [ENSMUST00000102721]	931.3	1108.9	1212.4	788.0	1232.3
37606	Slc2a4	Mus musculus solute carrier family 2 (facilitated glucose transporter), member 4 (Slc2a4), mRNA [NM_009204]	2671.4	3164.2	3896.8	2678.4	3514.8
37629	Slc26a3	Mus musculus adult male urinary bladder cDNA, RIKEN full-length enriched library, clone:9530095G06 product:solute carrier family 26, member 3, full insert sequence. [AK035716]	3.5	0.5	2.4	−0.6	0.7
37649	Slc39a6	Mus musculus solute carrier family 39 (metal ion transporter), member 6 (Slc39a6), mRNA [NM_139143]	394.0	458.7	495.7	337.4	439.4
37788	Slc25a25	Mus musculus solute carrier family 25 (mitochondrial carrier, phosphate carrier), member 25 (Slc25a25), mRNA [NM_146118]	463.5	675.5	383.3	882.4	239.2
37797	Slc34a2	Mus musculus solute carrier family 34 (sodium phosphate), member 2 (Slc34a2), mRNA [NM_011402]	12.0	10.8	23.8	31.7	33.6
37955	Slc2a12	Mus musculus solute carrier family 2 (facilitated glucose transporter), member 12 (Slc2a12), mRNA [NM_178934]	788.4	979.6	984.9	772.5	1079.8
38142	Slc6a5	Mus musculus solute carrier family 6 (neurotransmitter transporter, glycine), member 5 (Slc6a5), mRNA [NM_148931]	0.1	−0.1	−2.4	−2.7	−2.6
38146	Slc6a2	Mus musculus solute carrier family 6 (neurotransmitter transporter, noradrenalin), member 2 (Slc6a2), mRNA [NM_009209]	1.0	0.0	1.2	0.7	0.3
38165	Slc16a10	Mus musculus solute carrier family 16 (monocarboxylic acid transporters), member 10 (Slc16a10), mRNA [NM_028247]	354.3	259.7	450.7	269.8	272.6
38189	Slc27a5	Mus musculus solute carrier family 27 (fatty acid transporter), member 5 (Slc27a5), mRNA [NM_009512]	26.4	31.4	38.5	18.5	21.4
38257	Slc29a1	Mus musculus solute carrier family 29 (nucleoside transporters), member 1 (Slc29a1), mRNA [NM_022880]	1879.4	1814.8	2590.6	1555.4	1700.8
38352	Slc35b1	Mus musculus solute carrier family 35, member B1 (Slc35b1), mRNA [NM_016752]	12949.5	9672.3	13496.7	10980.4	12382.1
38377	Slc35c1	Mus musculus solute carrier family 35, member C1 (Slc35c1), transcript variant 1, mRNA [NM_211358]	12.7	5.8	9.0	7.5	7.6
38469	Slc5a12	Mus musculus solute carrier family 5 (sodium/glucose cotransporter), member 12 (Slc5a12), mRNA [NM_001003915]	13323.5	17818.5	15927.7	10891.2	14219.2
38473	Slc25a39	Mus musculus solute carrier family 25, member 39 (Slc25a39), mRNA [NM_026542]	72435.0	65549.7	83046.4	64404.7	77697.1
38510	Slc35a2	Mus musculus solute carrier family 35 (UDP-galactose transporter), member 2 (Slc35a2), mRNA [NM_078484]	241.3	221.4	250.3	241.7	252.3
38527	Slc25a34	Mus musculus 16 days neonate heart cDNA, RIKEN full-length enriched library, clone:D830027M14 product:Hypothetical mitochondrial energy transfer proteins, full insert sequence. [AK085913]	38.2	41.1	34.8	34.6	36.5
38605	Abca5	Mus musculus ATP-binding cassette, sub-family A (ABC1), member 5 (Abca5), mRNA [NM_147219]	197.1	235.6	243.0	213.2	256.9
38638	Slc26a6	Mus musculus solute carrier family 26, member 6 (Slc26a6), mRNA [NM_134420]	545.4	527.4	648.4	466.3	609.1
38656	Slc39a14	Mus musculus solute carrier family 39 (zinc transporter), member 14 (Slc39a14), mRNA [NM_144808]	1355.0	946.3	1051.5	1004.9	951.3
38716	Abce1	Mus musculus ATP-binding cassette, sub-family E (OABP), member 1 (Abce1), mRNA [NM_015751]	4628.9	4948.4	5905.1	4170.3	5699.8
38775	Slc45a3	Mus musculus solute carrier family 45, member 3 (Slc45a3), mRNA [NM_145977]	608.8	651.4	1019.9	678.4	756.3
38849	Abca7	Mus musculus ATP-binding cassette, sub-family A (ABC1), member 7 (Abca7), mRNA [NM_013850]	3386.4	3135.0	4446.1	3122.8	3517.2
38854	Slc6a6	Mus musculus solute carrier family 6 (neurotransmitter transporter, taurine), member 6 (Slc6a6), mRNA [NM_009320]	838.6	979.3	851.8	855.5	983.8
38869	Slco6c1	Mus musculus solute carrier organic anion transporter family, member 6c1 (Slco6c1), mRNA [NM_028942]	2.0	−0.7	−3.2	−0.5	−3.6
38880	Slc18a2	Mus musculus solute carrier family 18 (vesicular monoamine), member 2 (Slc18a2), mRNA [NM_172523]	29.1	33.8	29.3	16.1	25.9
38884	Slc25a21	Mus musculus solute carrier family 25 (mitochondrial oxodicarboxylate carrier), member 21 (Slc25a21), mRNA [NM_172577]	398.3	511.4	479.5	395.3	580.4
38918	Slc22a13	Mus musculus solute carrier family 22 (organic cation transporter), member 13 (Slc22a13), mRNA [NM_133980]	4211.3	4912.1	7134.3	6051.1	5895.5
38940	Slco1a5	Mus musculus solute carrier organic anion transporter family, member 1a5 (Slco1a5), mRNA [NM_130861]	4.2	−0.2	1.3	3.4	−0.3
38952	Slc35b2	Mus musculus solute carrier family 35, member B2 (Slc35b2), mRNA [NM_028662]	7304.1	8850.4	12555.7	7563.9	10492.0
39369	Slco5a1	Mus musculus solute carrier organic anion transporter family, member 5A1 (Slco5a1), mRNA [NM_172841]	−3.0	−0.8	−3.9	−1.7	−1.1
39383	Slc35f4	Mus musculus adult male testis cDNA, RIKEN full-length enriched library, clone:4930550L21 product:hypothetical protein, full insert sequence. [AK016089]	1.4	−2.2	−1.5	0.1	−0.5
39433	Slc25a44	Mus musculus solute carrier family 25, member 44 (Slc25a44), mRNA [NM_178696]	553.0	475.1	444.2	456.1	485.4
39591	Slc38a2	Mus musculus solute carrier family 38, member 2 (Slc38a2), mRNA [NM_175121]	4087.7	3079.5	3208.4	4124.7	3283.6
39592	Slc31a1	Mus musculus solute carrier family 31, member 1 (Slc31a1), mRNA [NM_175090]	30272.4	27447.8	28768.3	23173.2	27560.6
39597	Slc7a1	Mus musculus adult male bone cDNA, RIKEN full-length enriched library, clone:9830146A08 product:hypothetical protein, full insert sequence. [AK137576]	145.1	107.9	141.6	124.3	106.2
39624	Slc15a4	Mus musculus solute carrier family 15, member 4 (Slc15a4), mRNA [NM_133895]	3522.7	2823.1	4356.0	2927.5	2876.4
39689	Slc25a27	Mus musculus solute carrier family 25, member 27 (Slc25a27), mRNA [NM_028711]	53.0	48.1	62.2	53.8	46.1
39729	Slc30a3	Mus musculus solute carrier family 30 (zinc transporter), member 3 (Slc30a3), mRNA [NM_011773]	2.8	0.2	0.3	1.8	4.1
39730	Abcg5	Mus musculus ATP-binding cassette, sub-family G (WHITE), member 5 (Abcg5), mRNA [NM_031884]	17.9	24.4	28.7	14.9	28.0
39763	Slc12a4	Mus musculus solute carrier family 12, member 4 (Slc12a4), mRNA [NM_009195]	1198.3	1077.9	1342.6	1339.4	1290.6
39801	Slc24a3	Mus musculus solute carrier family 24 (sodium/potassium/calcium exchanger), member 3 (Slc24a3), mRNA [NM_053195]	47.0	54.0	78.2	41.3	63.1
39803	Slc2a8	Mus musculus solute carrier family 2, (facilitated glucose transporter), member 8 (Slc2a8), mRNA [NM_019488]	191.9	215.2	264.4	185.5	235.2
39833	Slc38a4	Mus musculus solute carrier family 38, member 4 (Slc38a4), mRNA [NM_027052]	24.1	22.6	23.1	27.4	30.6
39855	Slc22a8	Mus musculus solute carrier family 22 (organic anion transporter), member 8 (Slc22a8), mRNA [NM_031194]	13197.4	10919.2	13145.2	6697.8	7001.7
39902	Abcc1	Mus musculus ATP-binding cassette, sub-family C (CFTR/MRP), member 1 (Abcc1), mRNA [NM_008576]	617.3	891.6	1038.2	867.7	1014.1
39982	Slc2a4	Mus musculus solute carrier family 2 (facilitated glucose transporter), member 4 (Slc2a4), mRNA [NM_009204]	2577.0	3266.5	3798.4	2896.2	3548.9
40028	Slc25a42	Mus musculus solute carrier family 25, member 42 (Slc25a42), mRNA [NM_001007570]	213.8	188.8	205.7	289.0	222.5
40093	Slco4c1	Mus musculus solute carrier organic anion transporter family, member 4C1 (Slco4c1), mRNA [NM_172658]	53.5	144.8	267.1	223.1	252.2
40100	Slc4a11	solute carrier family 4, sodium bicarbonate transporter-like, member 11 [Source:MarkerSymbol;Acc:MGI:2138987] [ENSMUST00000099362]	13.1	19.1	44.1	39.9	39.5
40240	Abcb1b	Mus musculus ATP-binding cassette, sub-family B (MDR/TAP), member 1B (Abcb1b), mRNA [NM_011075]	191.6	284.6	217.3	1068.1	1300.6
40271	Slc35a5	Mus musculus solute carrier family 35, member A5 (Slc35a5), mRNA [NM_028756]	1133.1	1300.3	1313.8	932.4	1303.1
40350	Slc30a9	Mus musculus solute carrier family 30 (zinc transporter), member 9 (Slc30a9), mRNA [NM_178651]	769.3	904.6	644.0	1002.6	1007.7
40353	Slc7a13	Mus musculus solute carrier family 7, (cationic amino acid transporter, y+ system) member 13 (Slc7a13), mRNA [NM_028746]	30477.8	30984.5	29751.2	27109.8	30284.9
40418	Slc13a1	Mus musculus solute carrier family 13 (sodium/sulphate symporters), member 1 (Slc13a1), mRNA [NM_019481]	17591.3	16601.7	14889.7	11344.7	15336.2
40497	Abcg2	Mus musculus ATP-binding cassette, sub-family G (WHITE), member 2 (Abcg2), mRNA [NM_011920]	27874.8	29040.5	34021.8	24853.8	31006.7
40502	Slc44a4	Mus musculus solute carrier family 44, member 4 (Slc44a4), mRNA [NM_023557]	7058.1	6782.1	7773.0	6490.6	8712.4
40511	Slc7a12	Mus musculus solute carrier family 7 (cationic amino acid transporter, y+ system), member 12 (Slc7a12), mRNA [NM_080852]	170.9	221.3	218.1	89.1	156.1
40526	Slc43a2	Mus musculus solute carrier family 43, member 2 (Slc43a2), mRNA [NM_173388]	355.1	451.4	663.2	587.8	545.4
40608	Slc38a1	Mus musculus 12 days embryo embryonic body between diaphragm region and neck cDNA, RIKEN full-length enriched library, clone:9430020E10 product:hypothetical protein, full insert sequence. [AK162429]	17.0	21.5	27.6	16.0	20.9
40740	Slc25a33	Mus musculus solute carrier family 25, member 33 (Slc25a33), mRNA [NM_027460]	1103.3	997.6	782.6	794.1	1036.5
40807	Slc39a9	Mus musculus solute carrier family 39 (zinc transporter), member 9, mRNA (cDNA clone IMAGE:30536025), partial cds. [BC072655]	1796.6	1594.7	2021.4	1564.1	1816.2
40816	Slc25a26	Mus musculus solute carrier family 25 (mitochondrial carrier, phosphate carrier), member 26 (Slc25a26), mRNA [NM_026255]	1446.2	1707.4	1950.2	1149.2	1764.6
41084	Slco3a1	Mus musculus solute carrier organic anion transporter family, member 3a1 (Slco3a1), transcript variant 1, mRNA [NM_023908]	24170.3	22900.6	25768.2	23907.6	26723.1
41165	Slc17a1	Mus musculus solute carrier family 17 (sodium phosphate), member 1 (Slc17a1), mRNA [NM_009198]	32119.1	31174.1	34346.7	29392.1	33739.1
41224	Slc9a7	Mus musculus solute carrier family 9 (sodium/hydrogen exchanger), isoform 7 (Slc9a7), mRNA [NM_177353]	4.4	5.3	3.5	5.6	12.7
41317	Slc39a10	Mus musculus solute carrier family 39 (zinc transporter), member 10 (Slc39a10), mRNA [NM_172653]	803.4	1114.0	1116.8	1016.3	1362.3
41341	Slc9a3r2	Mus musculus solute carrier family 9 (sodium/hydrogen exchanger), isoform 3 regulator 2 (Slc9a3r2), transcript variant B, mRNA [NM_023449]	1188.2	1220.3	1503.9	1209.1	1184.3
41383	Slc2a4	Mus musculus solute carrier family 2 (facilitated glucose transporter), member 4 (Slc2a4), mRNA [NM_009204]	2876.1	3595.9	3881.4	3094.0	3990.4
41423	Abca9	Mus musculus ATP-binding cassette transporter sub-family A member 9 (Abca9), mRNA [NM_147220]	1.4	2.8	5.6	−0.8	3.6
41509	Slc22a16	Mus musculus solute carrier family 22 (organic cation transporter), member 16 (Slc22a16), mRNA [NM_027572]	100.4	88.1	146.3	81.6	87.4

41557	Slc25a40	Mus musculus solute carrier family 25, member 40 (Slc25a40), mRNA [NM_178766]	7.9	6.1	9.4	8.1	23.7
41633	Slc35f3	Mus musculus solute carrier family 35, member F3 (Slc35f3), mRNA [NM_175434]	143.8	241.2	239.0	152.6	287.0
41867	Slc44a4	Mus musculus solute carrier family 44, member 4 (Slc44a4), mRNA [NM_023557]	4994.9	4799.0	5313.5	4695.6	6041.3
41869	Abcc10	Mus musculus ATP-binding cassette, sub-family C (CFTR/MRP), member 10 (Abcc10), transcript variant mrp7A, mRNA [NM_145140]	16.7	23.1	22.4	15.3	19.3
42043	Slc4a3	Mus musculus solute carrier family 4 (anion exchanger), member 3 (Slc4a3), mRNA [NM_009208]	166.1	155.0	205.5	177.1	185.7
42059	Slc35b4	Mus musculus solute carrier family 35, member B4 (Slc35b4), mRNA [NM_021435]	2969.7	2882.2	3597.4	3082.4	3142.8
42226	Slc34a3	Mus musculus solute carrier family 34 (sodium phosphate), member 3 (Slc34a3), mRNA [NM_080854]	35887.8	36477.9	38994.3	28095.8	34654.7
42298	Slc7a6	Mus musculus 16 days neonate thymus cDNA, RIKEN full-length enriched library, clone:A130019A16 product:Similar to Y+L amino acid transporter 1, full insert sequence. [AK037437]	−2.3	0.7	−1.1	0.5	−0.2
42309	Abca16	Mus musculus ATP-binding cassette, sub-family A (ABC1), member 16 (Abca16), mRNA [NM_207130]	−1.6	−1.5	1.1	−0.5	−0.1
42393	Slc29a3	Mus musculus solute carrier family 29 (nucleoside transporters), member 3 (Slc29a3), mRNA [NM_023596]	552.9	537.6	1116.6	497.8	840.5
42403	Abca1	Mus musculus ATP-binding cassette, sub-family A (ABC1), member 1 (Abca1), mRNA [NM_013454]	1052.2	1702.3	1562.9	1063.7	1213.1
42478	Slc39a8	Mus musculus 10 days lactation, adult female mammary gland cDNA, RIKEN full-length enriched library, clone:D730033M24 product:similar to BCG INDUCED INTEGRAL MEMBRANE PROTEIN BIGMO-103 (UP-REGULATED BY BCG-CWS) [Homo sapiens], full insert sequence....	14.2	12.5	16.3	33.3	20.8
42583	Slc1a4	Mus musculus solute carrier family 1 (glutamate/neutral amino acid transporter), member 4 (Slc1a4), mRNA [NM_018861]	1080.8	1011.6	1243.4	1356.4	1483.9
42652	Abcc4	Mus musculus ATP-binding cassette, sub-family C (CFTR/MRP), member 4 (Abcc4), mRNA [NM_001033336]	277.1	332.0	298.9	241.0	253.4
42665	Slc6a11	Mus musculus solute carrier family 6 (neurotransmitter transporter, GABA), member 11 (Slc6a11), mRNA [NM_172890]	4.9	2.2	2.3	2.8	4.3
42684	Slc16a6	Mus musculus solute carrier family 16 (monocarboxylic acid transporters), member 6 (Slc16a6), transcript variant 1, mRNA [NM_001029842]	2495.1	2008.3	3879.1	3703.4	3081.4
42773	Slc26a11	Mus musculus solute carrier family 26, member 11 (Slc26a11), mRNA [NM_178743]	1866.1	2472.4	2827.4	1623.3	2347.9
42825	Slc2a9	Mus musculus solute carrier family 2 (facilitated glucose transporter), member 9 (Slc2a9), transcript variant a, mRNA [NM_001012363]	84.9	111.1	89.4	82.8	117.3
42973	Slc4a1ap	Mus musculus solute carrier family 4 (anion exchanger), member 1, adaptor protein (Slc4a1ap), mRNA [NM_009206]	1115.5	1101.8	1292.7	1019.0	1041.5
42990	Slc6a17	Mus musculus solute carrier family 6 (neurotransmitter transporter), member 17 (Slc6a17), mRNA [NM_172271]	79.6	100.5	165.5	106.5	111.2
43219	Abcc5	Mus musculus ATP-binding cassette, sub-family C (CFTR/MRP), member 5 (Abcc5), transcript variant 1, mRNA [NM_013790]	1121.4	1313.6	1344.1	997.6	1262.2
43229	Abcb11	Mus musculus ATP-binding cassette, sub-family B (MDR/TAP), member 11 (Abcb11), mRNA [NM_021022]	25.1	27.4	39.3	23.6	30.0
43271	Slc26a9	Mus musculus solute carrier family 26, member 9 (Slc26a9), mRNA [NM_177243]	3.1	2.9	4.7	5.1	6.8
43315	Slc16a6	Mus musculus solute carrier family 16 (monocarboxylic acid transporters), member 6 (Slc16a6), transcript variant 1, mRNA [NM_001029842]	203.6	207.2	267.7	374.8	354.6
43411	Slc25a40	Mus musculus 9.5 days embryo parthenogenote cDNA, RIKEN full-length enriched library, clone:B130008N03 product:unclassifiable, full insert sequence. [AK044862]	0.6	0.1	−1.2	1.1	0.0
43444	Slc25a3	Mus musculus adult male brain cDNA, RIKEN full-length enriched library, clone:3526402M16 product:solute carrier family 25 (mitochondrial carrier; adenine nucleotide translocator), member 3, full insert sequence. [AK028313]	665.7	611.3	723.9	717.4	532.4
43450	Slc35b3	Mus musculus 16 days neonate thymus cDNA, RIKEN full-length enriched library, clone:A130081B15 product:unclassifiable, full insert sequence. [AK079504]	52.6	49.3	62.6	55.5	58.7
43467	Slc10a7	Mus musculus solute carrier family 10 (sodium/bile acid cotransporter family), member 7 (Slc10a7), mRNA [NM_029736]	12.1	10.1	15.3	16.4	18.7
43505	Slc26a1	Mus musculus solute carrier family 26 (sulfate transporter), member 1 (Slc26a1), mRNA [NM_174870]	10240.4	8687.9	9911.3	9701.2	9446.5
43514	Slc5a8	Mus musculus solute carrier family 5 (iodide transporter), member 8 (Slc5a8), mRNA [NM_145423]	19978.3	30997.0	30890.8	27406.2	29279.8
43538	Slc22a19	Mus musculus solute carrier family 22 (organic anion transporter), member 19 (Slc22a19), mRNA [NM_144785]	2183.1	3238.0	4951.2	3877.3	4784.6
43596	Abcc5	Mus musculus ATP-binding cassette, sub-family C (CFTR/MRP), member 5 (Abcc5), transcript variant 1, mRNA [NM_013790]	326.9	343.9	340.3	267.9	353.8
43874	Slc22a5	Mus musculus solute carrier family 22 (organic cation transporter), member 5 (Slc22a5), mRNA [NM_011396]	2948.5	2956.5	2908.1	3496.7	2902.8
43956	Slc17a4	Mus musculus solute carrier family 17 (sodium phosphate), member 4 (Slc17a4), mRNA [NM_177016]	135.0	164.4	232.9	113.4	140.9
44040	Abca5	Mus musculus ATP-binding cassette, sub-family A (ABC1), member 5 (Abca5), mRNA [NM_147219]	36.2	54.2	47.3	58.3	68.1
44071	Abcf3	Mus musculus adult male pituitary gland cDNA, RIKEN full-length enriched library, clone:5330439H14 product:CDNA FLJ11198 FIS, CLONE PLACE1007697, WEAKLY SIMILAR TO GCN20 PROTEIN homolog [Homo sapiens], full insert sequence [AK030626]	731.7	699.8	832.6	719.5	772.2
44323	Slc22a3	Mus musculus solute carrier family 22 (organic cation transporter), member 3 (Slc22a3), mRNA [NM_011395]	0.1	4.4	2.9	2.1	2.5
44468	Slc1a4	Mus musculus solute carrier family 1 (glutamate/neutral amino acid transporter), member 4 (Slc1a4), mRNA [NM_018861]	765.0	778.5	1167.1	1023.0	1139.5
44490	Abca8b	Mus musculus ATP-binding cassette, sub-family A (ABC1), member 8b (Abca8b), mRNA [NM_013851]	29.2	25.8	22.7	17.6	22.2
44739	Slc9a6	Mus musculus mRNA for mKIAA0267 protein [AK122232]	637.4	774.7	841.3	765.0	874.1
44814	Slc9a3	Mus musculus adult male colon cDNA, RIKEN full-length enriched library, clone:9030624O13 product:SODIUM [AK033564]	5088.8	6286.6	4440.4	4266.8	5150.7
44856	Slc16a10	Mus musculus 6 days neonate spleen cDNA, RIKEN full-length enriched library, clone:F430003D17 product:solute carrier family 16 (monocarboxylic acid transporters), member 10, full insert sequence. [AK165240]	2146.4	1641.5	2060.1	1792.5	1526.1
45031	Abcb1b	Mus musculus ATP-binding cassette, sub-family B (MDR/TAP), member 1B (Abcb1b), mRNA [NM_011075]	561.1	672.7	626.9	2759.2	3354.2
45158	Slc16a1	Mus musculus solute carrier family 16 (monocarboxylic acid transporters), member 1 (Slc16a1), mRNA [NM_009196]	1561.6	1402.0	3405.8	2094.3	1640.0

Table S2. Microarray analysis for expression of transporter in mouse kidney.

Gene name	Description, distribution and substrate	Fold Change
Transporters expressed at levels significantly more than 1.5-fold that of male wild-type mice.		
Abcb1b	ATP-binding cassette, sub-family B,	4.68
	Ovary, female kidney	
	Estron sulfate, estradiol	
Slc22a7	organic anion transporter2	1.80
	Kidney, liver	
	Glutarate (K_m=15.8 μM)	
Slc6a15	Neurotransmitter transporter,	1.65
	Brain, kidney	
	Neutral amino acid (e.g. proline)	
Transporters expressed at levels significantly less than 1.5-fold that of male wild-type mice.		
Slc22a8	Organic anion transporter 3	0.56*
	Brain, kidney, liver	
	para-aminohippurate (K_m=65 μM)	
Slc7a12	Cationic amino acid transporter, y^+ system	0.61*
	Female kidney	
	No data	

Table S3. Sequence identity between human, monkey, rat and mouse ABCB1

Species	Protein sequence identity (%)
Human ABCB1	100%
Monkey ABCB1	96%
Rat Abcb1b	81%
Mouse Abcb1b	81%

Table S4. Non-conserved amino acids in human ABCB1

	Human ABCB1	Monkey ABCB1	Rat ABCB1b	Mouse ABCB1b
Position	Amino acid			
24	E	K	K	K
635	V	I	I	I
759	A	V	V	V
851	F	L	L	L
1003	I	V	V	V
1027	M	K	K	K
1038	G	N	N	N
1103	R	Q	Q	Q
1168	K	R	R	R

Figure S1

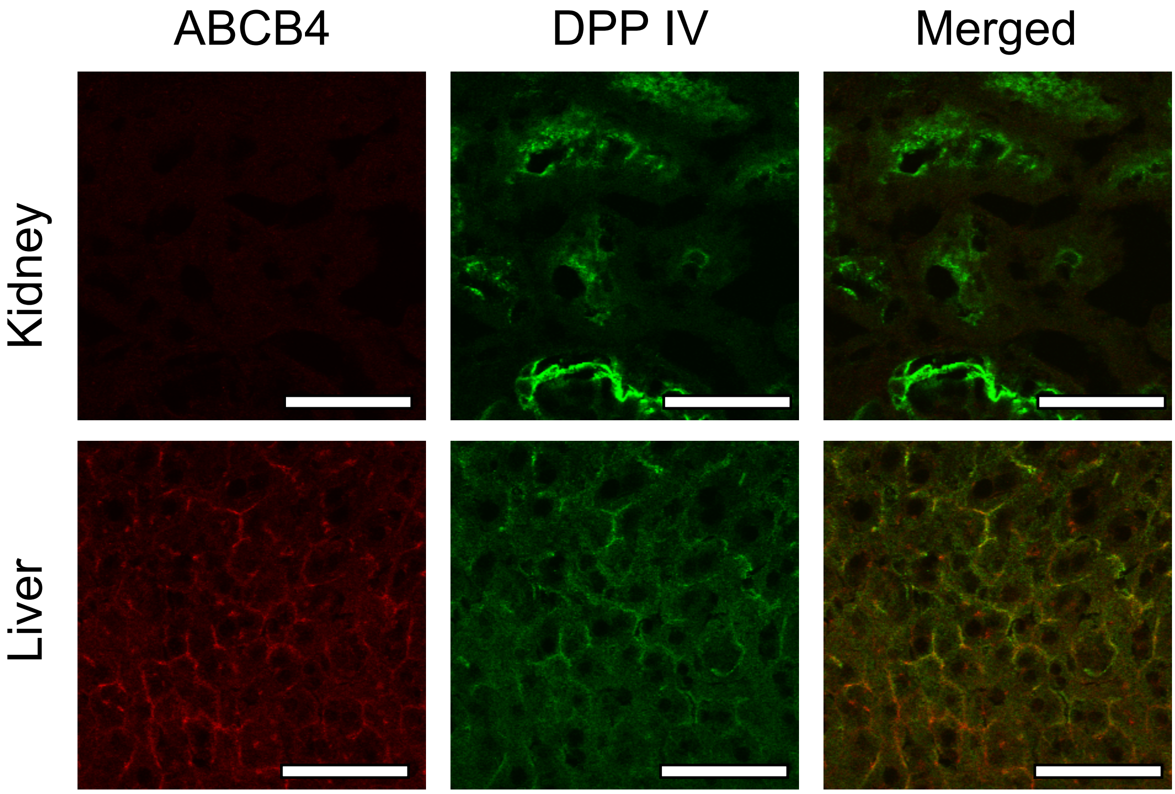

Figure S1. Immunohistochemical analysis of ABCB4 expression in wild-type mouse kidney and liver. Dipeptidyl peptidase IV (DPP IV) is a marker of apical membranes. To detect ABCB4 and DPP IV, fluorescent conjugated secondary antibody (Alexa Fluor 594 or Alexa Fluor 488) was applied. Representative immunoblots are shown. Scale bars, 50 μm.
